# Carbon-Related Materials: Graphene and Carbon Nanotubes in Semiconductor Applications and Design

**DOI:** 10.3390/mi13081257

**Published:** 2022-08-04

**Authors:** Mohammadreza Kolahdouz, Buqing Xu, Aryanaz Faghih Nasiri, Maryam Fathollahzadeh, Mahmoud Manian, Hossein Aghababa, Yuanyuan Wu, Henry H. Radamson

**Affiliations:** 1 School of Electrical and Computer Engineering, College of Engineering, University of Tehran, Tehran 1439957131, Iran; 2 Institute of Microelectronics, Chinese Academy of Sciences, Beijing 100029, China; 3 University of Chinese Academy of Sciences, Beijing 100029, China; 4 Research and Development Center of Optoelectronic Hybrid IC, Guangdong Greater Bay Area Institute of Integrated Circuit and System, Guangzhou 510535, China; 5 Department of Electronics Design, Mid Sweden University, Holmgatan 10, 85170 Sundsvall, Sweden

**Keywords:** graphene, CNTs, nano biosensors, photodetectors, CNTFET, graphene FET

## Abstract

As the scaling technology in the silicon-based semiconductor industry is approaching physical limits, it is necessary to search for proper materials to be utilized as alternatives for nanoscale devices and technologies. On the other hand, carbon-related nanomaterials have attracted so much attention from a vast variety of research and industry groups due to the outstanding electrical, optical, mechanical and thermal characteristics. Such materials have been used in a variety of devices in microelectronics. In particular, graphene and carbon nanotubes are extraordinarily favorable substances in the literature. Hence, investigation of carbon-related nanomaterials and nanostructures in different ranges of applications in science, technology and engineering is mandatory. This paper reviews the basics, advantages, drawbacks and investigates the recent progress and advances of such materials in micro and nanoelectronics, optoelectronics and biotechnology.

## 1. Introduction

The carbon nanotube history simply extends back to the time when the carbon fibers were investigated and prepared by Thomas A. Edison. He used an early model of carbon fibers as a light bulb’s conductive filament [1]. Further studies on carbon fibers took a long time while finally the research led to applying the vapor growth technique for manufacturing the carbon filament [2,3]. Later, intensive effort resulted in the synthesis of high-quality carbon filaments. Roger Baken utilized a DC arc discharge to grow a “graphite whisker” in a chamber at 92 atm of argon at a temperature of 3900 K. The final whisker product had diameters in the range of 1–5 μm and lengths up to 3 cm. The distinct characteristics of whiskers such as flexibility, tensile strength, Young’s modulus and conductivity at room temperature were investigated [4]. In a parallel study, Bollmann and Sprendborougli investigated the lubricating properties of rolled graphite [5].

Research on carbon crystal growth proceeded and led to the growth of thin carbon tubes [6,7]. Reports of these carbon tubes inspired Kroto and Smalley to discover fullerenes [8]. Afterward, Huffman improved the synthesis method of fullerenes [9]. These carbon assemblies consist of 60 atoms in which each carbon atom has *sp^2^* bonds. Smalley and Kroto were awarded a Nobel Prize in 1985 for their discovery.

Later, Sumio Ijima introduced a real breakthrough in carbon nanotube study in 1991 when he presented carbon nanotubes as a member of the fullerene family. This material is a rolled graphene sheet into an unsealed tube on both ends [10]. Initially, in these investigations, multi-walled carbon nanotubes (MWCNTs) were grown by using a simple arc-discharge method. In these experiments, single-walled carbon nanotubes (SWCNTs) were synthesized as byproducts and were not really under control until 1997 [11]. SWCNT synthesis was then found to be more controllable by using a similar process of producing MWCNT but in the presence of transition metal particles [12,13].

Later, graphene was experimentally obtained by Geim and Novoselov in 2004 [14]. The idea was based primarily on isolating 2D crystals of carbon, *sp*^2^ bonds, and provided a new view to the phenomena in quantum physics. In other respects, carbon crystals without *sp*^3^ bonds could be formed with stable *sp*^2^ bonding. Their work was awarded the physics Nobel Prize in 2010.

Carbon, as the most versatile element with diverse forms of bonding, can produce many structures, such as 0D, 1D, 2D and 3D. Carbon with *sp*^2^ hybridization can form a hexagonal-shaped lattice typical of a sheet of graphite, while carbon with *sp*^3^ hybridization can construct the most complex 3D crystalline structure in diamond [15]. Fullerene, carbon nanotubes and graphene are examples of 0D, 1D and 2D structures, respectively. They are generally known as carbon nanomaterials.

For a long time, they have been used in a variety of devices in microelectronics [16,17,18]. A small quantity of carbon has been used in bipolar transistors to impede the out-diffusion of boron in the SiGe base [19]. The other applications relate to the increase in the thermal budget in silicide formation [20,21,22,23] or to the decrease in the intermixing of Si into the Ge or SiGe interface [24]. For photonic application, carbon has been widely used for bandgap engineering in SiGe for IR detection [24,25,26,27,28]. Meanwhile as an individual element, carbon may be considered in a 3D crystal as diamond and as 2D crystals or nanotubes for devices [29].

Graphitic materials are often used in the description of characteristics of carbon-based materials such as graphite, nanotubes, large fullerenes, etc. [30]. Graphene is a 0.34 nm-thick monolayer graphite. A 2D film of *sp*^2^-bond carbon atoms is arranged into a hexagonal lattice [31,32,33,34]. Graphene is also regarded as either unrolled SWCNTs or a large atomic plane taken from graphite [35]. Thus, it is important to understand the structures and characteristics of graphene.

**Figure 1:**
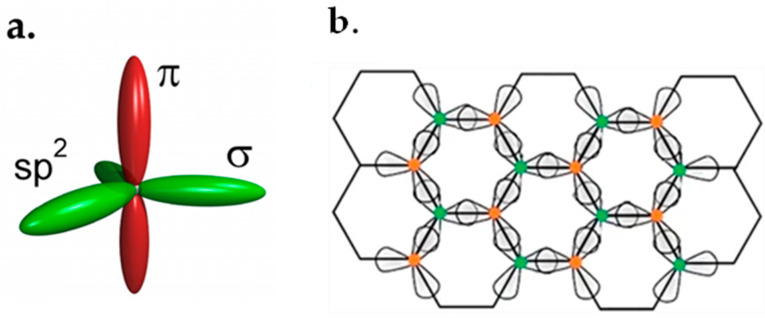
(**a**) *sp*^2^ hybrids of each carbon atom [36]. (**b**) *sp*^2^ hybrids of carbon atom connecting with neighboring ones in graphene [15].

Figure 1a demonstrates *sp*^2^ hybrids of each carbon atom in graphene. These hybrids form *σ* bonds with a length of 1.42 Å, which connect the neighboring carbon atoms [36]. These *σ* bonds result in excellent mechanical features. A pi bond (π bond) is a bond formed by the overlap of orbitals in a side-by-side fashion. The *π* orbitals orthogonal to the hexagonal plane of graphene are responsible for the graphene’s electronic features and energy bands [37,38]. Figure 1b illustrates how *sp*^2^ hybrids of each carbon atom connect with its neighboring atoms.

Carbon nanotubes are graphene layers rolled around a cylinder with a very high ratio of length to diameter. CNT has multiple helicities and chirality types compared to a rolled graphene film. CNTs with their unique properties are potentially useful in various applications. The main research of CNTs involves the improvement of synthesis techniques and the study of their properties for novel applications [39,40,41,42]. CNTs can be utilized in many fields such as energy conversion electrode structures, sensor and biosensor design, gas discharge tubes for telecommunication, screening of electromagnetic waves, batteries, hydrogen storage and composite materials [43,44,45]. One of the most critical issues in CNTs is the high surface-to-volume ratio. This feature refers to concentrating the whole weight in its surface layer and makes it a proper candidate for electrochemical and adsorption applications [46,47,48]. In other words, high sensitive surface structure for adsorption and great electronic properties make CNTs advantageous for miniaturized sensors and biosensors [49,50]. Furthermore, significant works have been undertaken which revealed many structural, mechanical, electrical, electrochemical and chemical properties of CNTs which inspired scientists to employ these unique cylindrical molecules in an extensive variety of applications.

CNTs are categorized depending on the number of graphene layers. There are two types of carbon nanotubes: single-walled CNTs (SWCNTs) and multi-walled CNTs (MWCNTs). In order to simplify the concept of physical structure, SWCNTs are cylindrical tubes of 0.5–1 nm diameter capped by hemispherical ends. They can be assumed to be a rolled or folded graphene sheet [51]. MWCNTs comprise several concentric cylinders of typically 2–100 nm in diameter with a layer spacing of 0.3–0.4 nm. The interlayer interval between graphene sheets in graphite is approximately equal to the distance between the MWCNT interlayer; hence, the MWCNT can be considered as a folded graphite sheet [52,53].

## 2. Physical Structures

Carbon nanotubes are fullerene-related molecules composed of graphene sheets rolled into a cylindrical tube [54,55]. Hence, the surface-to-volume proportion is very large and in many applications, this high ratio is the advantage of nanotubes [56]. The aspect ratio is based on the synthesis method.

Graphene is an carbon allotrope which has two dimensions with hexagonal lattices [57]. In the graphene lattice plane, three outer-shell electrons of each atom occupy three hybrid *sp*^2^ orbitals. These orbitals form *σ* bonds with the nearest atoms. The remaining outer-shell electron occupies the *p*_z_ orbital which is perpendicular to the lattice plane. The *p*_z_ orbitals hybridize together to form two half-filled bonds of free-moving electrons, π and π*, which are responsible for the electrical and transport properties of graphene [58,59,60]. In the case of small CNTs, the overlap of inner π bonds is much greater, contributing to the structural properties, whereas in planar graphene there is much less overlap between orbitals.

**Figure 2:**
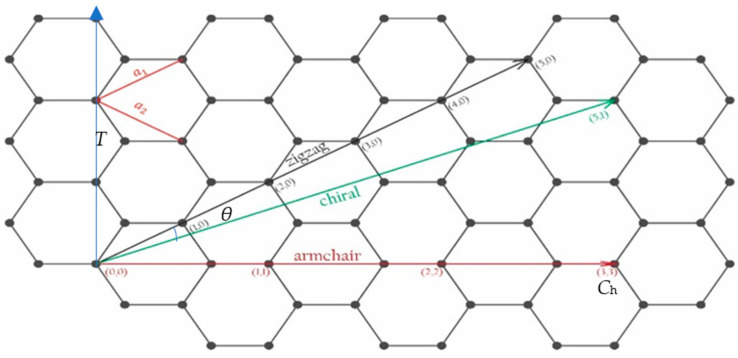
Armchair, chiral and zigzag types of nanotube structures.

As mentioned before, a CNT can be treated as a rolled graphene sheet. Any CNT can be specified using three geometrical specifications called chiral vector (***C_h_***), the chiral angel (*θ*) and translation vector (***T***) [53], as illustrated in Figure 2. ***C_h_*** is represented by a pair of indices ($n_{1},n_{2}$) which defines the way the graphene sheet is wrapped. It can be calculated as:

(1) $$C_{h}=n_{1}a_{1}+n_{2}a_{2}\;\mathrm{or}\;C_{h}=\left(n_{1},n_{2}\right)$$

where

(2) $$a_{1}=\left(\sqrt{3}\frac{a}{2}.\frac{a}{2}\right),a_{2}=\left(\sqrt{3}\frac{a}{2}.-\frac{a}{2}\right),a=2sin\frac{\pi }{3}a_{c-c}=2.461\;\dot{A}$$

***T*** is the smallest vector that is perpendicular to ***C****_h_*. One could say that ***T*** indicates the periodicity of the CNT lattice along the tube axis. Equation (3) defines this property.

(3) $$T\cdot C_{h}=0$$

*θ* specifies the direction of ***C_h_***. In other words, *θ* is the angle between ***a***_1_ and ***C_h_*** (Equation (4)) [61].

(4) $$cos\theta =\frac{a_{1}\cdot C_{h}}{\left|a_{1}\right|\cdot \left|C_{h}\right|}=\frac{2n_{1}+n_{2}}{2\sqrt{n_{1}{}^{2}+n_{1}n_{2}+n_{2}{}^{2}}}$$

CNTs have three possible structures depending on the way they are wrapped from graphene sheets: armchair, zigzag and chiral. The armchair and zigzag nanotubes have a chiral vector of (*n*, *n*) and (*n*, 0), respectively [61,62]. While chiral CNTs have a chiral angle between 0 and π⁄6 [63].

The length of the chiral vector, |***C_h_***|, is patently equal to the nanotube circumference. Hence, one could acquire the radius of the nanotube using Equation (5).

(5) $$r_{nanotube}=\frac{|C_{h|}}{2\pi }=a\frac{\sqrt{n_{1}{}^{2}+n_{1}n_{2}+n_{2}{}^{2}}}{2\pi }$$

### 2.1. Tight-Binding Approximation

The potential problems of a solid system with an enormous number of particles can be solved using the linear summation of atomic orbitals or tight-binding approximation [64]. It is known from Bloch’s theory that electron wave function is described through the linear combination of isolated atom Hamiltonian eigenstates. The variational principle is utilized to minimize the error in this method. In order to acquire a better level of accuracy, the orbital overlap integrals (orbital overlap integral indicates the correlation of the two wave function orbitals) can be considered for the nearest neighbor orbitals, second neighbor orbitals, third neighbor orbitals, etc. [64,65].

The accuracy of the tight-binding approximation is between empirical methods and ab initio methods. Hence LCAO (Linear Combination of Atomic Orbitals) can be considered as a semi-empirical technique.

### 2.2. Graphene Band Structure

**Figure 3:**
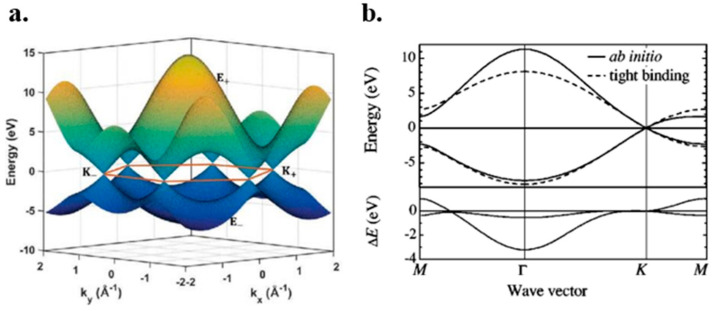
(**a**) Nearest neighbor tight-binding approximation of graphene. Γ, M and K are the high symmetry points of graphene lattice. (**b**) The comparison between expensive and accurate ab initio study of graphene and its NNTB model approximation [66].

Graphene *σ* bonds are confined near the nuclei; hence, the only considered bonds in the graphene band structure are *π* and π* bonds. The following equation specifies the NNTB (nearest neighbor tight bonding) model of graphene [60,66,67].

(6) $$Ε\left(k\right)=\pm \frac{\gamma_{0}\sqrt{1+4cos\left(\frac{a\sqrt{3}k_{x}}{2}\right)cos\left(\frac{ak_{y}}{2}\right)+4{cos}^{2}\left(\frac{ak_{y}}{2}\right)}}{1\pm s\sqrt{1+4cos\left(\frac{a\sqrt{3}k_{x}}{2}\right)cos\left(\frac{ak_{y}}{2}\right)+4{cos}^{2}\left(\frac{ak_{y}}{2}\right)}}$$

where $\gamma_{0}$ is the orbital overlap integral ($\int \hat{H}\varphi^{*}{}_{i}\left(r-T\right)\varphi_{j}\left(r\right)dr$) and s can be determined using $s=\int \varphi^{*}{}_{i}\left(r-T\right)\varphi_{j}\left(r\right)dr$. In most cases, *s* is minor enough to be neglected, especially in the band structure calculation of nanotubes [66]. Figure 3a illustrates the nearest neighbor tight-binding calculation for graphene.

In the proximity of the Dirac points, the energy dispersion of graphene is linear. In some cases, such as the electron transport, graphene band structure can be treated as double cones connecting at the zero bandgap points [58]. The comparison between ab initio study (which is expensive and accurate) of graphene and its NNTB approximation is depicted in Figure 3b.

### 2.3. From Graphene to Nanotubes

A simple but effective technique to describe the single-walled nanotube band structure is the zone folding approximation. When graphene sheet is folded into a nanotube, the boundary condition is:

(7) $$\psi_{n}\left(R\right)=\psi_{n}\left(R+C_{h}\right)$$

From Bloch’s theorem, we can conclude:

(8) $$K\cdot C_{h}=2\pi m$$

In this method, by applying Born-von Karman boundary conditions along the circumferential direction, the allowed electron states are limited. The electronic band structure of nanotubes can be derived by substituting permissible ***K*** lines from Equation (8) into the graphene energy dispersion equation [12,68,69,70,71,72].

### 2.4. Bandgap

SWCNTs are completely metallic if *n*_1_ − 2*n*_2_ = 0 or *n*_1_ = *n*_2_. This is while SWCNTs could be a narrow gap semiconductor if *n*_1_ − 2*n*_2_ = 3*k*, where *k* is an integer, and are semiconducting in other cases. The results of bandgap calculations for zigzag nanotubes using different methods are collected in Table 1.

## 3. Electrical and Material Properties

### 3.1. Semi-Metal Characteristics

**Figure 4:**
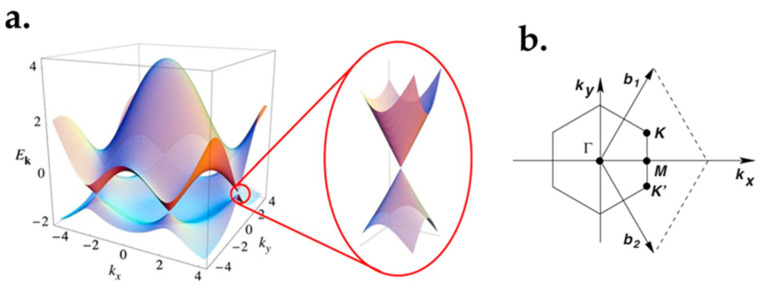
(**a**) Graphene’s band structure. (**b**) First Brillouin zone of the graphene lattice [60].

Zero bandgap is one of the most impressive characteristics of graphene, resulting from its valence band meeting the empty conduction band at six points (see Figure 4a). Specifically, two carbon atoms in each graphene unit cell will lead to two conical points (K and K’) for each Brillouin zone during the bands crossing (Figure 4b). As the bandgap determines transistors’ on and off region, a small bandgap leads to a low on–off ratio. As a result, graphene’s zero band gap hinders its applications in devices such as field-effect transistors (FETs) [76]. Another disadvantage of a zero bandgap is that bi- and multilayer graphene are non-luminescent unless a bandgap was introduced [77]. Accordingly, it should be noted that graphene FETs are not suitable for digital applications and logic circuits [30,78].

Many approaches have been applied to tune the graphene’s bandgap, such as adding an external electric field, slicing the graphene sheets into thin ribbons and chemical doping [31,79,80]. Generally, introducing various atoms/molecules to the graphene’s surface can substantially alter its properties. It is worth noting that the modification of graphene’s surface is now creating a wide portfolio of newly synthesized graphene derivatives with tunable optical, chemical and mechanical properties. Two of the most studied graphene derivatives are graphene oxide and fluorographene [81]. Bilayer graphene also has zero bandgap [82]. By adding an external electric field, a charge density would appear at its surface. It leads to a shift of the Fermi level, which in turn creates a bandgap [29,83]. This bandgap is tunable between 0 and 0.3 eV, which lies in the mid-wave infrared range [82]. However, no Fermi level shift would happen when applying an electric field to a monolayer graphene, since the monolayer graphene’s band structure and bandgap differs from that of bilayer graphene [84].

A finite gap can be created by implementing narrow ribbons of graphene. The bandgap is affected by the ribbon’s width and orientation [31]. These ultranarrow graphene nano-ribbons (GNRs) have properties similar to those of semiconductors. The origins of the band gaps for GNR with armchair or zigzag shaped edges vary. The former originates from quantum confinement, and edge effects play a crucial role. For the latter, the band gaps arise from a staggered sublattice potential due to spin ordered states at the edges. First-principles calculations confirmed that the GNR’s bandgap is inversely proportional to the width of the ribbon, due to the quantum confinement and edge effects [85]. For instance, Barone et al. predicted that the ribbon’s width should be lowered to 2–3 nm to obtain a bandgap like that of Ge or InN [86]. Correspondingly, to obtain larger bandgaps like those of InP, Si or GaAs, the ribbon’s width should be reduced to approximately 1–2 nm. Reaching such dimensions has yet been impossible [86,87,88]. GNR FETs at room temperature have a bandgap of 100 meV [89]. The chemical doping approach shifts the Fermi level of graphene. This charge doping varies the symmetric potential [90]. Other methods, for example, interaction with gases and defect generation are also used to tune the bandgap. It is even reported that water molecules adsorbed on graphene’s surface can introduce a bandgap of 0.206 eV [76].

Linear dispersion is one of the characteristics of graphene materials, as illustrated in Figure 4a. The holes and electrons at the Dirac point are in linear relations of energy-momentum dispersion. The Dirac point is located close to the charge-neutrality point. Graphene behaves similar to a massless Dirac fermion, because of the two similar carbon sub-lattices in the graphene’s crystal structure [91]. Thus, graphene’s electrical features can be described using the Dirac’s equation [91,92,93,94,95].

Graphene also has strong ambipolar electric field effect characteristic. Different from ordinary metals, its charge carriers can be tuned from electrons to holes by applying an external electric field. It can be either p-type or n-type depending on the electric field [31]. In other words, the carrier type is not pre-defined. This is because when graphene’s energy is above 0, its current states are electron-like and have negative charges [91].

### 3.2. Electrical Conductivity and Ballistic Transport

Graphene’s mobility is extremely high. Three macroscopic forces, namely drift, diffusion and drag force, can affect its mobility. Drift force is caused by the electrostatic field. Diffusion force is caused by carriers’ random motion, and drag force is because of the carriers scattering in the lattice [78]. There are four scattering mechanisms which impact graphene’s mobility. They are caused by charged impurities, surface roughness, optical phonons and acoustic phonons. It has been experimentally confirmed that the charged impurities have the dominant effect on the mobility. Thus, mobility at room temperature can be increased by reducing the charged impurity concentration. This can be obtained by annealing or using high-κ dielectric materials [96].

It is reported that graphene’s carrier mobility is between 1 and 20 × 10^3^ cm^2^/V·s. It should be noted that higher mobilities have been reported for clean graphene that is suspended or placed on boron nitride [15]. The charge density has to be lower for gaining higher mobilities. The best obtained mobility at room temperature was 15,000 cm^2^/V·s [20,97,98].

Compared to silicon and III-V semiconductors, graphene has much higher intrinsic carrier mobility [34,37,91,99]. Therefore, graphene has a rapid electronic transport which benefits high-frequency applications for transistors [79,100].

Graphene has high electrical conductivity due to its high mobility [101]. The conductivity can be changed when adsorbing different atoms and molecules such as K and NH_3_. If the absorbed species are weakly attached, graphene’s high conductivity remains because the absorptions may act as acceptors or donors. While other absorbed species, for example H^+^ or OH^−^, create a mid-gap state close to the graphene’s neutrality point, which decreases the conductivity of graphene. Therefore, graphene can be transformed into an insulator by exposing to other materials such as H_2_ [102,103]. Scattering mechanisms, such as extrinsic, intrinsic and impurity scattering, can also be used to change graphene’s conductivity [99].

**Figure 5:**
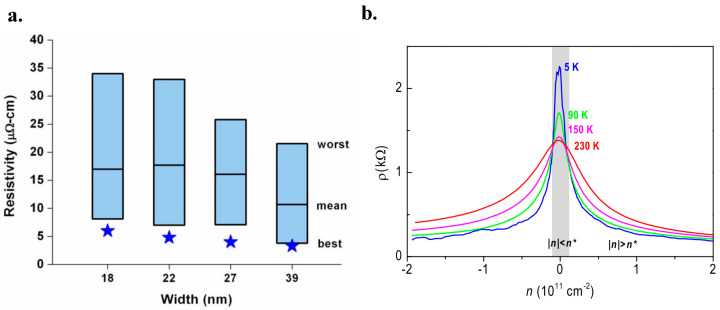
(**a**) The resistivity of individual GNRs with different linewidth. For each line-width, the best, worst and mean values of the resistivity are displayed. At each width, 10 data points corresponding to 10 GNRs. The equivalent Cu resistivity is shown as a star [99]. (**b**) The resistivity of graphene is a function of temperature and carrier density [33].

Graphene has higher resistivity than that of Cu wires, as demonstrated in Figure 5a. Accordingly, GNRs have lower conductivity. It is published that the mean resistivity of a GNR with a line-width of 18–52 nm is three times larger than that of a Cu wire with a similar width [99,104].

Like any other semiconductors, graphene’s resistivity is affected by temperature variations. Graphene has a mobility of 170,000 cm^2^/V·s near 5 K, because the carriers have a large mean free path at low temperature and a near-ballistic transport. Figure 5b illustrated that the resistivity behaves in two different ways according to the carrier density as temperature increases. When the carrier densities are higher than 0.1 × 10^11^ cm^−2^, the graphene displays a metallic conductivity feature. Specifically, the increased temperature results in a larger resistivity. The mobility reaches about 120,000 cm^2^/V·s at about 240 K, which is higher than any discovered semiconductors. While it displays a non-metallic feature at low densities (|*n*| < 0.1 × 10^11^ cm^−2^), indicating that the resistivity increases with the temperature decreasing. Low density occurs close to the charge neutrality point [3].

Ballistic transport is another remarkable characteristic of graphene [28]. Diffusive and ballistic phonon transport are two particular kinds of thermal transport. If the channel length is longer than the carrier mean-free path length, the phonons may be scattered during the transport process. Thermal transport is diffusive. Otherwise, the thermal transport is ballistic [105]. Thus, graphene is a choice for applications when both ballistic and diffusive transport regimes are required [106].

### 3.3. Melting Point and Stability

Non-electrical features of graphene are still not fully discovered. As an example, the melting temperature of graphene has not been found yet, even though we know that the melting temperature of ultra-thin films decreases as the thickness decreases [101]. It is even reported that graphene would not melt because the atomic vibration amplitudes close to the equilibrium position are smaller than interatomic distances [91].

Young’s modulus, flexibility and thermodynamic stability are a few extraordinary properties of graphene. Graphene exhibits remarkable elastic stiffness with a Young’s modulus up to 1.0 TPa, which occurs near a strength number of 120 GPa (100 times higher than that of steel) [97,103,107]. Graphene could be stretched about 20% more than any known crystals. Such high flexibility makes graphene suitable for flexible electronics [104,108,109,110].

Thermodynamical stability is another unique feature of graphene. It is known that ultra-thin films are thermodynamically unstable as the thickness decreases. As for graphene film, experimental research showed that it is thermodynamically stable and conductive even at nanometer ranges [28]. Thus, the thermodynamical stability benefits the scaling of graphene devices and makes it an alternative material for the applications of integrated circuits (IC) [34,111].

### 3.4. High Transparency

**Figure 6:**
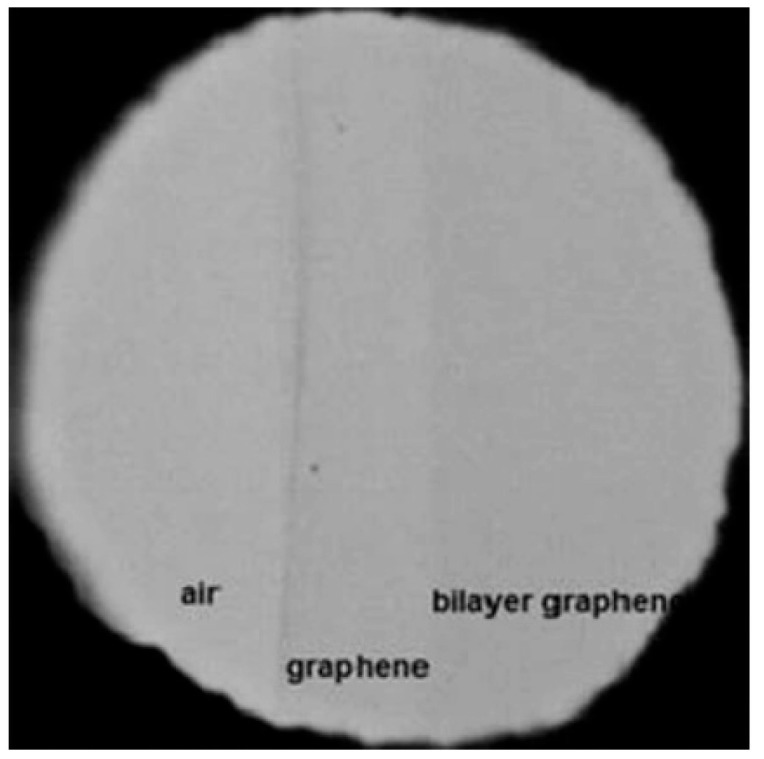
Transparentness of air, graphene and double-layer graphene [112].

Graphene can absorb a notable fraction of incident light, although it has one atomic layer [112]. The optical absorption is in proportion to the number of layers. Lower than 1% of the visible light is reflected, which in the case that the quantity of layers rises to 10, it grows to 2%. Approximately 2.3% white light is absorbed by each layer. Additionally, graphene has high transparency and its transmittance gets lowered when the number of layers gets higher [94,97,112,113,114], see Figure 6. It can be concluded that the more layers the graphene has, the less transparent it exhibits [112]. Optical absorption is a function of wavelength in graphene. In the near-IR region at around 1 eV (1240 nm), 2.3% absorption is observed. Accordingly, the absorption will be larger when at energies larger than 1.5 eV (corresponding to 825 nm wavelength) [105,112].

### 3.5. Thermal Conductivity

The material’s structure affects the thermal conductivity, and it becomes crucial when electronic devices are scaled down to nanometers. It is reported that carbon allotropes possess a wide range of thermal conductivity [115]. Amorphous carbon material has the lowest value of about 0.01 W/mK, whereas diamond possesses a high value of around 2000 W/mK [105]. It has been theoretically proven that thermal conductivity can be extremely large in 2D and 1D crystals. It is reported that graphene has a thermal conductivity of around 5300 W/mK at room temperature [30,37,101,104,106,116,117].

The high κ parameter is also an extraordinary feature of graphene. It determines how well the graphene conducts heat. By using edge roughness, this parameter can be altered [105].

## 4. Growth and Transfer of Graphene

**Figure 7:**
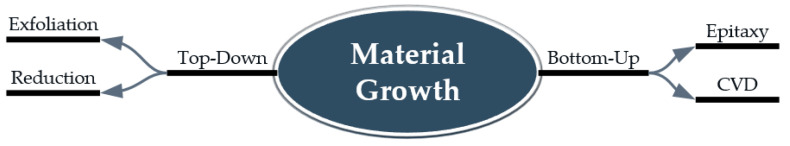
Schematic of graphene growth classifications.

The most crucial step in the fabrication of microelectronic devices is material growth. However, if the aim is to obtain a monolayer, this step gets more crucial. In this part, several methods of graphene growth on different substrates are reviewed. Additionally, the advantages and disadvantages are given. Graphene growth methods are divided into two categories, top-down method and bottom-up method (see Figure 7).

### 4.1. Top-Down Method

Pre-deposited or synthesized carbon-based materials, such as graphite or graphite oxide, are used to grow graphene. Many studies have demonstrated various processes either physical or chemical to produce graphene. Exfoliation and reduction will be discussed here as the prime approach to achieve the high-quality graphene for electronic applications [118].

#### 4.1.1. Exfoliation

Exfoliation is a simple and high-yielding approach to obtain mass production of graphene, although not fully purified [119,120]. Graphene is obtained by decreasing graphite’s number of monolayers [121,122,123]. A few achievable options of exfoliation, such as Scotch tape, drawing method and graphite sonication, will be discussed in the following paragraphs.

**Figure 8:**
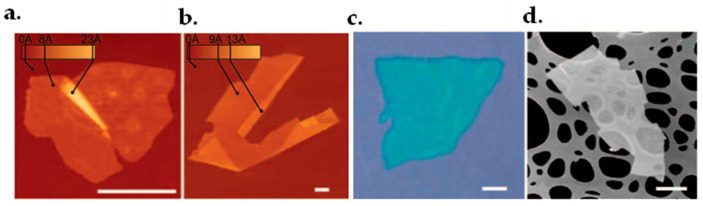
Monolayer 2D crystallites. (**a**) NbSe_2_ by atomic force microscopy (AFM), (**b**) graphite by AFM, (**c**) Bi_2_Sr_2_CaCu_2_O_x_ by SEM and (**d**) MoS_2_ seen by optical microscope [124].

The Scotch-tape and drawing method [124] is known as a mechanical exfoliation to produce graphene. It was firstly developed by Novoselov and Geim. With an adhesive tape, the graphene layers are recurrently peeled off from graphite crystals. The optically transparent flakes stuck to the tape are dissolved in acetone for detachment. After a few more steps, the flakes, containing monolayers of graphene, are then sediment on a silicon wafer. By using a dry deposition method in the drawing procedure, the floating stage could be avoided. However, the drawback of this method is that the obtained graphene cannot be scaled further and the size is limited to a few micrometers [125]. It should be mentioned that the Scotch-tape and drawing method is not limited to producing graphene but can also be used to produce some other thin layer materials such as freestanding atomic planes of boron nitride, mica, dichalcogenides and complex oxides. Figure 8 shows the morphology of some thin layer materials obtained with such method.

**Figure 9:**
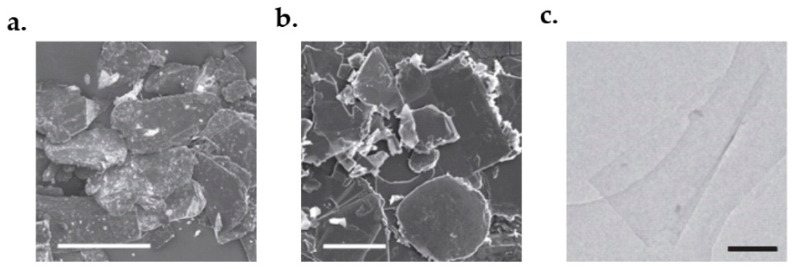
(**a**) SEM image of sieved raw graphite. (**b**) SEM image of sediment after centrifugation. (**c**) Bright-field TEM images of single-layer graphene flakes with a deposition from GBL [126].

Graphite sonication [126] is another method used to obtain high-quality graphene. It starts with a dispersion of sieved graphite powder, see Figure 9a. A grey liquid containing many macroscopic aggregates and a homogeneous phase would be obtained after sonication, see Figure 9b. Using mild centrifugation, the aggregates would be removed. Moderate levels of sedimentation and aggregation take place after three weeks of centrifugation. However, high-quality dispersions would remain for at least five months. The graphite dispersion is then passed across polyvinylidene fluoride filters (PVDF) to find its concentration. After this process, a few five-layer graphene flakes and large amounts of monolayers will be obtained in a g-butyrolactone (GBL) solvent, as shown in Figure 9c.

**Figure 10:**
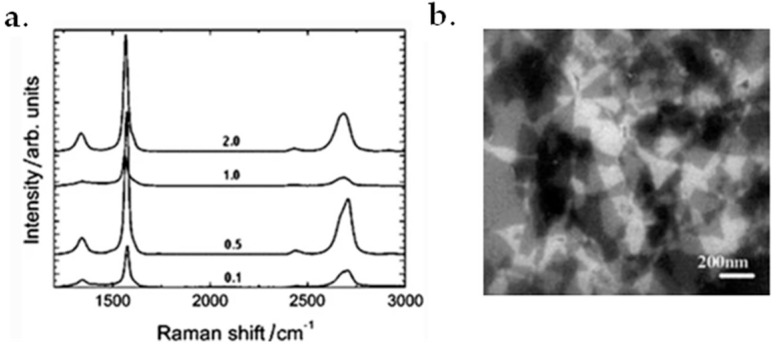
(**a**) Raman spectra of the obtained graphene using the sonication method with 0.1, 0.5, 1.0 and 2.0-wt% graphite in HMIH [128]. (**b**) SEM result of exfoliated graphite oxide [129].

Graphite flakes can also be used as raw materials to produce graphene with the sonication method [127,128]. Firstly, various amounts of graphite flakes would be mixed with 5 g 1-hexyl-3-methyl-imidazolium hexafluorophosphate (HMIH) by grounding in a mortar for 10 min. Secondly, the mixture would be placed in a tubular plastic reactor and set in an ultrasonic bath at room temperature for 0.5, 6.5, 14.5 and 24 h, respectively. Figure 10a shows the Raman spectra of the obtained graphene sheets, which were collected from different wt% of dispersed graphite in HMIH. At last, the mixtures were centrifuged at 4000× *g*RPM (revolutions per minute) for 30 min and the grey to black liquid phase containing graphene would be obtained. A concentration of 5.33 mg/mL was obtained by sonicating 3.5 mL of dispersed graphite for 24 h [128].

Another promising sonication method was proposed by Wang et al. In their method, the primary material was firstly dispersed in water under ultrasonication for 30 min, followed with the centrifugation process at 4000 rpm for another 30 min. Then, the obtained product was dried under a vacuum. The obtained product was then suspended in water again using ultrasonication for 2 h. The final step was to further remove aggregates by centrifuging at 10,000 rpm for 15 min. Figure 10b shows the SEM result of the final products [129]. The products have a high conductivity of about 550 S/cm. Moreover, the obtained films have high transparency up to 70% with a thickness of 1000–3000 nm.

“Defect-free” graphene has been recently synthesized by employing a unique flow-aided sonication exfoliation method [130]. Mitlin et al. discovered a critical and unexpected relationship between the graphene’s chemical/structural defectiveness and the ability to suppress the growth of dendrites, branch-like filament deposits on the electrodes, which may penetrate the barrier between the two halves of the battery.

#### 4.1.2. Reduction

##### Pyrolysis

**Figure 11:**
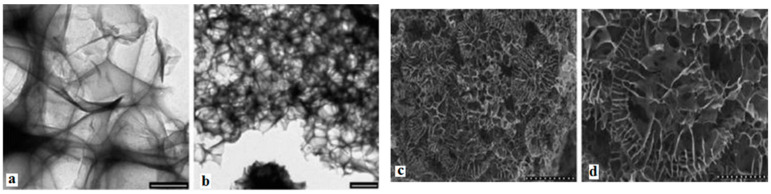
TEM (**a**,**b**) and SEM (**c**,**d**) results of the as-synthesized graphene sheets [131].

Another method to synthesize graphene is pyrolyzing sodium ethoxide. The main idea is reducing ethanol via sodium metal, thermal decomposition of ethoxide material and finally rinsing off the sodium salts with water. To do this, 2 g of sodium and 5 mL of ethanol (the molar ratio is 1:1) would be sealed in a reactor vessel and heated at 220 °C for 72 h. The obtained product, namely graphene precursor, was then rapidly thermal decomposed and washed with 100 mL deionized water. The mixture was then filtered and dried in a vacuum at 100 °C for 24 h. The yield graphene concentration is about 0.1 g/mL. The obtained products are displayed in Figure 11a,b. The SEM images of the products are shown in Figure 11c,d [131].

##### Reduction with Graphite Oxide

**Figure 12:**
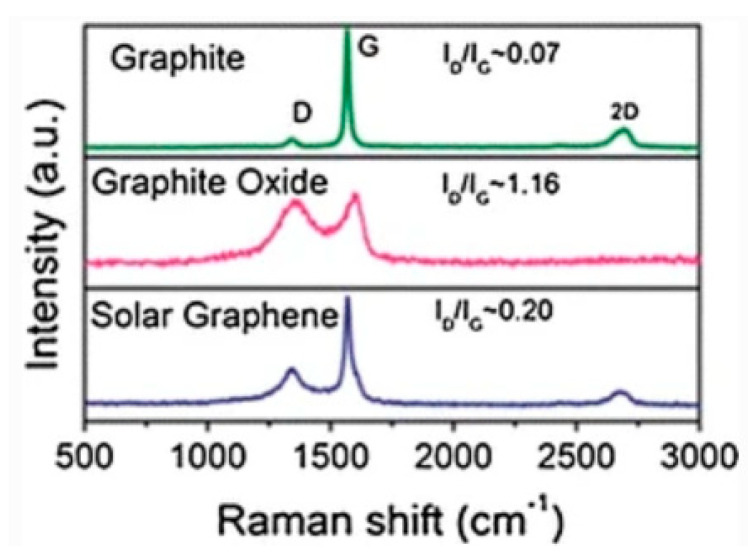
Graphite’s Raman spectroscopy, GO and solar exfoliated graphene [27].

Another reduction method to produce graphene is by rapid heating graphite oxide (GO) [132,133]. A small amount of graphene flakes could be obtained with the reduction of GO films. The reduction could be done either by hydrazine or by annealing in an argon/hydrogen atmosphere, which is thermal reducing [134]. The former is called chemically reduced graphene oxide (CRGO) and the latter is called thermally reduced graphene oxide (TRGO). Nevertheless, graphene obtained with these methods has a lower quality compared to that of the mentioned Scotch-tape method. There are many ways to get reduced graphene [135]. For example, converging the solar radiation is one of the ways to obtain a fast temperature increase at a rate of more than 100 °C/s. The Van Der Waals force within GO layers can be overcame with the introduction of pressure during the radiation process. Thus, the layers can be reduced. Figure 12 displays the Raman spectra comparison of graphite, GO and solar graphene. Compared to graphite, the GO has a high and wide D band at 1368 cm^−1^ and a wide and right-shifted G band at 1604 cm^−1^. Such features might be caused by graphite oxidation, which would lead to a reduction in the in-plane *sp*^2^ domains. After the reduction process, the G band of the obtained solar graphene was shifted back and was matched with that of graphite. By analyzing the intensity ratio between the D band and G band (*I_D_/I_G_*), the GO had a ratio of 1.16, indicating a large number of defects. As for solar graphene, the lowest ratio of 0.20 indicates a high quality of the obtained product [27]. For the chemical reduction method, the first step would be exfoliating the GO in water ultrasonically to obtain ~1 nm-thick GO films, followed by a chemical reduction treatment by hydrazine. Due to the hydrophobic features, the products would aggregate and precipitate [136]. The obtained graphene would not be monolayers, but with a thickness of approximately 30–100 nm.

Graphite dioxide can also be used in the chemical reduction method. In this method, magnesium is oxidized in a reaction with CO_2_: 2 Mg(s)+CO_2_(g) → 2 MgO(s) + C(s). Chakrabarti obtained several graphene layers with a length of 50–300 nm. In this approach, Mg was put in a bowl with dry ice and covered with a dry ice slab. The Mg burned and yielded black products. The products were then soaked into HCl acid, where the Mg and MgO reacted with HCl and the byproduct was MgCl_2_ which would dissolve in water. At last, the cleaning and drying process is completed to achieve several graphene layers [137].

#### 4.1.3. Others

Other top-down methods have also been reported to produce graphene, such as unzipping nanotubes [138,139,140], super acid de-solvation [141], arc-discharge [142], and reduction of sugars, such as glucose, fructose and sucrose [143].

### 4.2. Bottom-Up Method

The above-mentioned methods are mostly used in laboratories other than industry. To obtain high-quality graphene industrially, the products should be controllable and reproducible in both layer thickness and size. While the bottom-up method is introduced for massive production of high-quality graphene. In the method, carbon sources are decomposed and stored in the catalyst layer. This continues until the solved carbon reaches its saturation degree. Then, precipitation of carbon atoms occurs either from catalyst’s top or bottom side depending on the catalyst, and then graphene layers begin to grow. In this part, the procedures of growing graphene on various substrates will be reviewed.

#### 4.2.1. Epitaxial Method

High conductivity, high transparency and controllability over the number of the final layer are crucial for producing high-quality graphene. Epitaxy is considered to be one of the most promising methods to obtain high-quality graphene with a layer thickness of less than 10 nm. Many conditions and approaches can be used in graphene epitaxial growth. Some of them will be reviewed below for a better understanding of the essential procedures.

**Figure 13:**
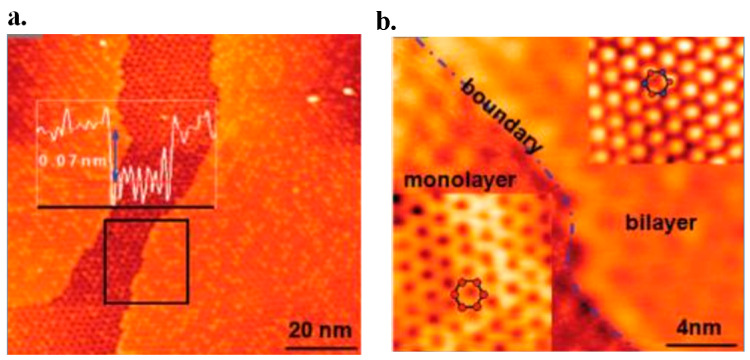
(**a**) STM result of the single-layer graphene (dark region) and double-layer graphene (the bright regions); (**b**) HRSTM image. Hexagon with red dots stands for the lattice of the single layer, and hexagon with red and blue dots stands for two non-identical triangular sublattices of the double-layer graphene [145].

Graphene can be epitaxially grown on substrates such as *6H-SiC* (0001) or *4H-SiC* (0001) [144]. A two-step method was proposed by Huang et al. to grow graphene on an n-type Si-terminated 6*H-SiC* (0001) substrate. In this method, the SiC is firstly decomposed with high-temperature annealing, which leads to the desorption of Si from the surface and accumulation of carbon atoms. By annealing at a temperature of 1200 °C or higher (Figure 13), monolayers, bilayers, trilayers and even thicker graphene films could be grown on top of the substrate. It should be noted that the pressure should be at 5 × 10^−9^ mbar in the chamber [145].

**Figure 14:**
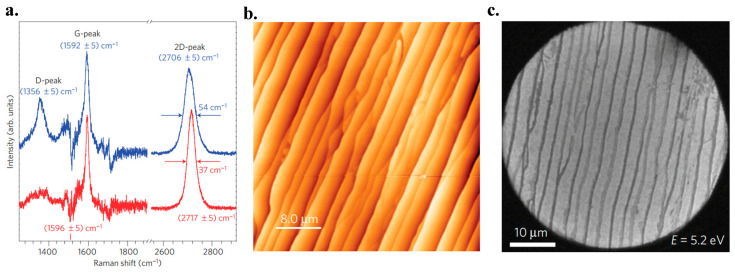
(**a**) Raman spectra of epitaxial graphene obtained in condition of UHV (blue) and Ar flow (red). (**b**) AFM surface morphology of graphene on *6H–SiC* (0001) which was annealed in Ar. (**c**) LEEM surface morphology of graphene obtained in conditions similar to (**b**) [146].

High-quality graphene sheets a few hundred nanometers in size were achieved with the method. The graphene has a mobility of 2000 cm^2^ V^−1^ S^−1^ at 27 K. There is no need to transfer the graphene layers to another insulating material, since the underneath SiC substrate is an insulator. Nevertheless, the quality of the grown films is poor due to the lack of uniformity and continuity. It is reported that small graphene domains (30~100 nm in diameter) could be obtained on the Si (0001) substrate, whilst extended domains of 200 nm in diameter are achieved on the C-faced (0001) substrate during the ultrahigh vacuum (UHV) annealing process. To remove defects of the surface, the substrates were firstly etched with hydrogen. Then, the growth process was undertaken in a cold-walled reactor vertically. The rate of heating and cooling was restricted to 2~3 °C/s and the growth time was 15 min. Because of the wide range of temperature (1500~2000 °C) and pressure (10~900 mbar), a small Ar flow during growth was proved to have much better results compared to UHV. Figure 14a illustrates the Raman spectra of graphene obtained by epitaxy in both UHV and Ar flow conditions. The lower amplitude of the D-band peak in the figure in Ar flow condition indicates a larger domain size of graphene [135]. Figure 14b,c shows the surface morphology of the epitaxial graphene obtained in the Ar flow circumstances taken by AFM and low energy electron microscopy (LEEM). The LEEM image reveals the macro-terraces covered with graphene up to 50 μm long and at least 1 μm wide [146].

**Figure 15:**
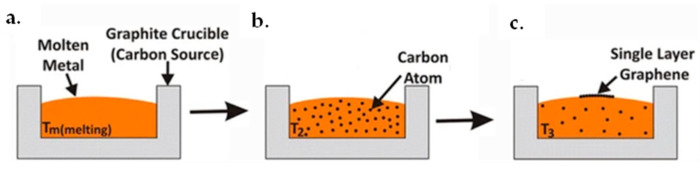
(**a**) Nickel metal melts in a graphite crucible at *T_m_*, (**b**) carbon atoms dissolve into the liquid nickel at temperature *T_2_* and (**c**) graphene precipitates at the top of the liquid nickel at *T_3_* [147].

**Figure 16:**
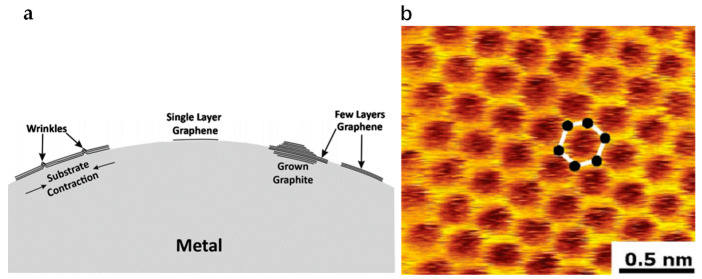
(**a**) Schematic of wrinkle, monolayer graphene and multilayer graphene [147]. (**b**) STM result of monolayer graphene [148].

Amini et al. reported a different method using molten metal–carbon solutions [147]. Single-layer or multilayer graphene could be achieved with this method from the molten solutions. The first step of this procedure would be dissolving carbon in the molten metal. Subsequently, graphene growth would start in the decreased temperatures at the surface of the molten metal. A schematic diagram of this procedure is illustrated in Figure 15. Metal such as nickel melts with a carbon source. The carbon source could be graphite powder, a piece of carbon or graphite crucible which holds the metal solution, see Figure 15a. The interaction between molten nickel and graphite crucible would dissolve carbon and saturates it in the molten nickel, see Figure 15b. Afterward, with temperature decreasing, carbon solubility reduces and excess carbon atoms would precipitate on the solution surface, as illustrated in Figure 15c. Arc melting inside a resistance furnace was chosen for the dissolution procedure. During the process, the chamber was firstly vacuumed to 10^−6^ Torr and then backfilled with purified argon twice. The melting process was completed at a current of 75 amps for 20 s. When the temperature reached 1500 °C, the samples were held at the temperature for 16 h and then cooled down to room temperature. The temperature changing rate was kept at 10 °C/min. When using Cu as the molten metal, the melting process was carried out in a graphite crucible as a carbon resource. A hypereutectic composition of Ni + 2.35 wt% C was chosen, and a certain quantity of chunk carbon was added to the molten metal. The possible morphologies of the obtained graphene are schematically illustrated in Figure 16a.

Although many studies were proposed by employing single crystalline SiC as the substrate, Hofrichter et al. reported that amorphous SiC is also an alternative substrate to gr graphene. They successfully controlled the carbon concentration of the solution by using a 50 nm SiC layer together with a 500 nm Ni layer. Rapid annealing at 1100 °C was undertaken to dissolve SiC in Ni. The advantage is that vacuum is not mandatory during the annealing process. When the temperature decreases, carbon atoms accumulate and precipitate at the Ni surface, forming graphene layers. To transfer the obtained graphene film to the desired substrates, nitric acid and hydrogen peroxide were used for etching Ni and dissolving Si. Afterward, the floating graphene was transferred to an insulating substrate, for example, SiO_2_. STM results demonstrate that the graphene obtained by Hofrichter et al. is of high quality [148], as shown in Figure 16b.

#### 4.2.2. Chemical Vapor Deposition Method

With the above-discussed methods, it is still challenging to obtain high-quality large-size graphene. Chemical vapor deposition (CVD) [149,150] is a possible approach which can be used for massive production of graphene. The idea behind it is chemically decomposing carbon-related materials such as ethanol, acetylene, methane or methanol on catalysts surfaces such as nickel [151,152,153,154] or copper [155,156,157,158,159].

**Figure 17:**
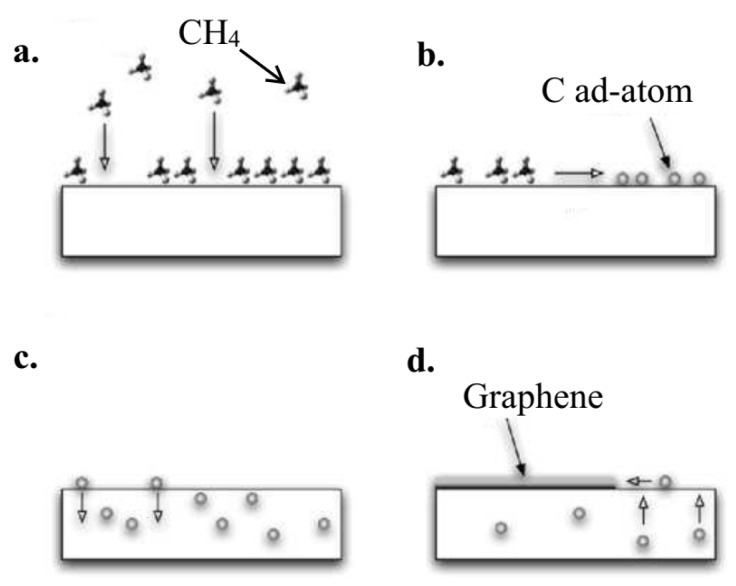
(**a**) Ni surface adsorbing methane. (**b**) Methane atoms getting pyrolyzed. (**c**) Ni-carbon solution forming. (**d**) Carbon atoms precipitating and forming graphene [160].

Ni is the most commonly used catalyst substrate in the CVD growth procedure of graphene. CVD growth of graphene is a four-step procedure which is similar to the growth method of CNTs. Figure 17 demonstrates a typical CVD growth procedure. To start, carbon-related materials were transferred to the Ni surface. Then, the materials decomposed into carbon ad-atoms which dissolved into the Ni substrate. By lowering the substrate temperature, the saturation occurred and carbon atoms precipitation onto the Ni surface forming a graphene layer [160].

Ni has proven to be an excellent substrate to grow CNTs, though it has drawbacks when used to growth graphene. It would create grains in multilayer graphene at the Ni grain boundaries. Additionally, the more the carbon atoms dissolved, the more layers the graphene precipitates on the Ni surface [155].

**Figure 18:**
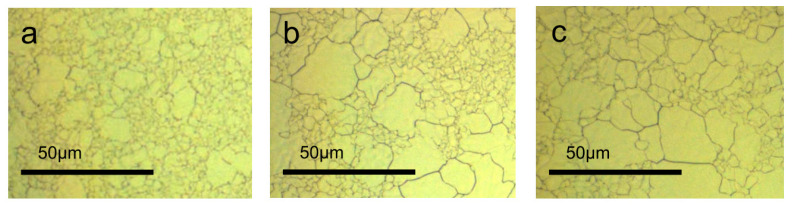
Optical image of Ni films annealed at 1000 °C with different times, (**a**) 1 min, (**b**) 15 min and (**c**) 40 min [161].

To get high-quality graphene films with Ni substrate, the grain sizes of Ni film must be increased. Thiele et al. reported that the grains of the Ni film would prefer to orientate in the (111) direction by reducing the internal stress. Additionally, this procedure also enlarges the grain size. Figure 18 displays the grain size of Ni films annealed at 1000 °C at different times [161].

In order to get rid of those drawbacks, Cu was selected as an alternative substrate during the CVD growth process. Carbon has a lower solubility in Cu even at high temperatures up to 1000 °C in Ar or H_2_ atmosphere. Therefore, the low concentration of dissolved carbon has little effect on the graphene thickness. The graphene film forms directly on the Cu substrate. Thus, it is easier to control the growth process compared to that of Ni [160,162]. Heat treatment could also enlarge the Cu grains, therefore, cause better coverage. Li et al. reported that more than 95% coverage of single-layer graphene was obtained on a 25 μm Cu substrate at 1000 °C by using a mixture of methane and hydrogen. The obtained graphene film has a size of 1 by 1 cm. Additionally, they discovered that bi- and trilayer flakes grown on the remaining 5% surface would not grow larger with time [155].

Reducing the thermal budget is one of the challenges in the CVD growth method. Nandamuri et al. reported that graphene films can be grown at low temperatures between 650 and 700 °C using nickel as the catalyst substrate when the methane was changed to acetylene [163]. An alternative method to grow graphene at low temperatures was using plasma-enhanced chemical vapor deposition (PECVD) [164]. Malesevic et al. reported that four to six atomic layer graphene sheets were grown at 700 °C by controlling the recombination of carbon radicals in a microwave plasma without using catalysts on the surface [165]. However, one of the disadvantages of the PECVD method is that it is difficult to grow large-area graphene, because the large electrical field is perpendicular to the surface which prevents the graphene’s planar growth. It leads to the vertically grown graphene sheets [158,166,167].

It is reported that less-layer with large-area graphene films were grown on Ni substrate by direct-current (DC) PECVD method. By applying a DC voltage, the density of defects in the obtained graphene films could be minimized. High-volume hydrogen was used to enhance the graphene uniformity and present the formation of CNTs during the process. In this method, Ni film was first exposed to hydrogen at a flow rate of 100 sccm (standard cubic centimeters per minute) under 1 Torr of pressure for 30 min. Then, the temperature was increased to 1000 °C, and simultaneously plasma was added at 500–800 V. Next, the film was exposed to C_2_H_2_ gas at 2 sccm under the same pressure for 10–20 min. At last, the samples were cooled down to room temperature [168].

A similar study using radiofrequency PECVD also was carried out to grow large-area graphene at 650 °C on Ni films deposited on SiO_2_/Si substrates [169]. Firstly, a 150 nm Ni film was deposited on SiO_2_/Si surface using direct-current magnetron sputtering. Then, the Ni films were placed inside the PECVD chamber and the pressure was lowered to 37.5 mTorr. The films were heated at 650 °C for 40 min with H_2_ at a pressure of 1.5 Torr. Later, plasma was applied at 13.56 MHz and 650 °C. A mixture gas of CH_4_, Ar and H_2_ was applied at the same time with a flow rate of 2, 80 and 40 sccm, respectively. The chamber pressure was maintained at 7.5 Torr for 30 s. At last, the samples temperature was cooled down at 10 °C/s in an Argon ambient. Graphene was achieved as a result of carbon segregation. The residual Ni film was then etched away using a 1 M FeCl_3_ aqueous solution.

As a summary, Table 2 gives a comparison of those aforementioned graphene preparation methods. CVD can produce good-quality graphene for electronic applications. However, the practical application of graphene is still hindered by the high price and insufficient supply.

### 4.3. Graphene Film Transfer

**Figure 19:**
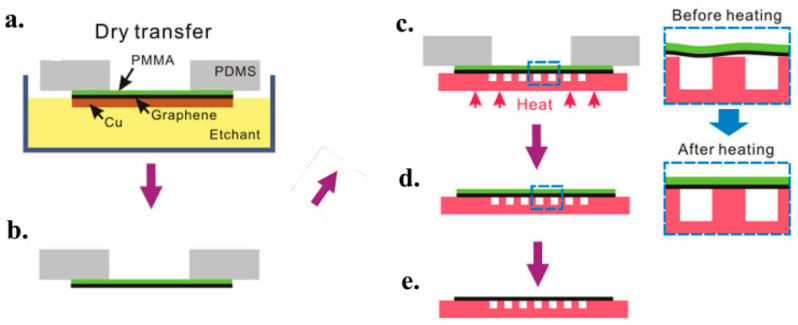
Illustration of a dry transfer procedure on shallow substrates. (**a**) The copper is etched with the PDMS/PMMA/graphene floating over the etchant. (**b**) The sample is rinsed and dried with the PDMS “handle”. (**c**) The PDMS/PMMA/graphene is placed onto the target substrate and heated. (**d**) Peel off the PDMS block. (**e**) The PMMA is thermally removed in a furnace at 350 ℃ with Ar and H_2_ for 2 h. The heat treatment in c makes the rough PMMA/graphene film fully contact with the substrate, as shown in the magnified views of figure (**c**,**d**) [170].

During the fabrication process of electronic devices, it is crucial to transfer the grown graphene film onto the desired position. Graphene transfer methods have been reviewed in recent articles [153,170,171,172,173,174]. Those methods could be classified into dry and wet categories. The chosen type is based on graphene’s state after etching. If the graphene adheres to the substrate, the dry transfer method is preferred. Alternatively, if at a floating state, it is better to choose a wet transfer method. In general, it would cause fewer contamination and damage to the graphene if the transfer has fewer steps using liquids. Figure 19 demonstrates a dry transfer procedure schematically.

Many epitaxial approaches are used to grow multilayers graphene on flat substrates, such as using thermal release tape, a bilayer gold film and polyimide. The obtained graphene can be peeled off with the dry transfer method. It is reported that a polydimethylsiloxane (PDMS) frame can be used to transfer graphene/PMMA film from the copper etchant [170]. Followed with a PMMA heat treatment, the adhesion between the graphene and the substrate layer can be enhanced. The heating process softens the PMMA layer. Hence, the gap between the substrate and the graphene is reduced.

**Figure 20:**
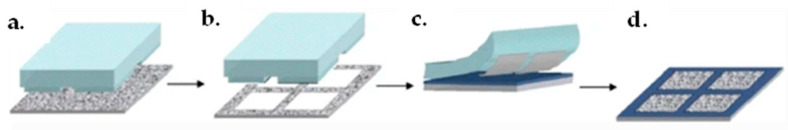
Transfer printing method. (**a**) Material deposition on a glass surface, (**b**) inking of the PDMS stamp, (**c**) contacting the inked stamp and a heated Si/SiO_2_ substrate and (**d**) the stamp is peeled off to achieve the deposited material [171].

Figure 20 illustrates an alternative dry transfer method utilizing a PDMS stamp. The initial step is spin coating hydrazine suspensions on a substrate of oxygen plasma-treated glass. It has to be noted that the mixture of hydrazine suspensions and the speed of spin-coating influence the density of the deposited films. The second step is contacting the glass substrates and PDMS. The third step is contacting the inked stamps with the Si/SiO_2_ substrates. The contacts take a few days at room temperature to get a complete transfer. The final step is removing the stamp from the Si/SiO_2_ [171].

Gold is also usable in the dry method for removing graphene which is strongly adhered to a silicon substrate [172]. That is to say, the gold film can be used as a transfer stamp to peel off graphene from the HOPG surface. Initially, graphene patterns are fabricated on the HOPG surface with photolithography and an O_2_ plasma etching process, followed with gold deposition. Next, the gold film is peeled off with thermal release tape. Next, the graphene patterns are transferred and pressed onto the desired preheated substrate. Finally, the tape is removed and the gold film is etched away with a gold etchant solution.

**Figure 21:**
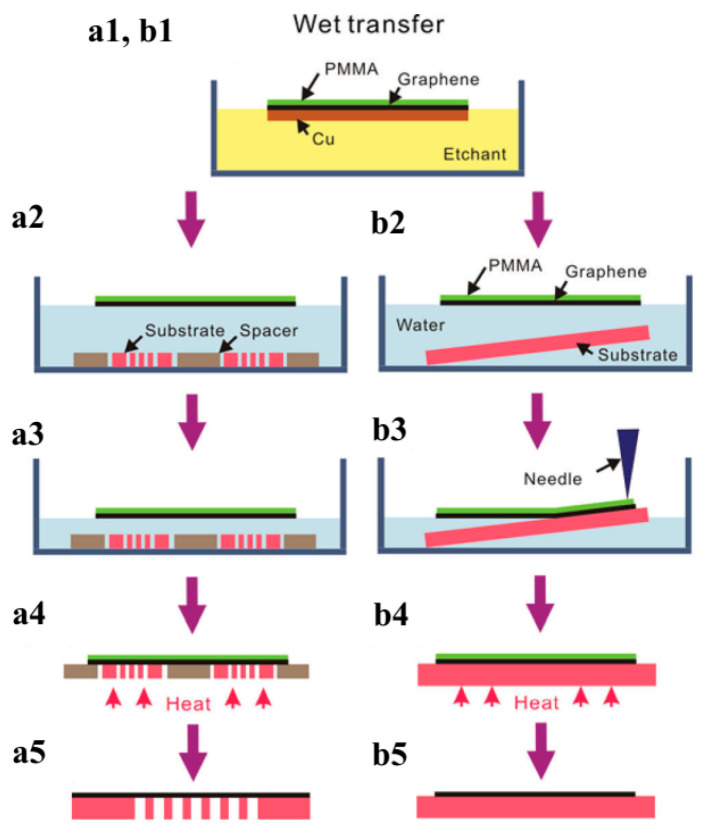
Illustration of a wet transferring procedure on perforated substrates: **a1**–**a5** and flat substrates (**b1**–**b5**). **a1**,**b1**, the copper foil is etched away. **a2**, multiple substrates with spacers were diped in Hexamethyldisilazane (HMDS) to fully support the graphene/PMMA membrane. **b2**, substrate is placed in the water at an inclined angle. **a3**, HMDS was removed. **b3**, the PMMA/graphene film is lowered onto the substrate by removing water with a syringe. **a4**, the critical point drying was used. **b4**, the dried PMMA/graphene/substrate is heated to remove the PMMA. **a5**,**b5**, the PMMA is removed with acetone [170].

Figure 21 displays a wet transfer procedure. A thin PMMA film is spin-coated on the graphene surface. The ferric chloride etchant is used to etch Ni and Cu foil, and then the graphene/PMMA film is rinsed with deionized water. An annealing process will enlarge the caliber of wet transfer onto the substrates [170]. The heating process can decrease the graphene sheet’s resistance. It is reported that the resistance of the graphene sheet grown with the CVD method can be decreased by a factor of 2 with annealing treatment. Accordingly, the graphene films produced by CVD and transferred with the wet method are more suitable for the fabrication of electronic devices such as transparent conductive films [175,176].

## 5. Growth and Mechanisms of CNTs

### 5.1. Growth

Controlling nanotubes’ properties has been facilitated by investigation on new synthesis methods over the years. Many aspects of fundamental research and practical applications have been motivating the synthesizing approaches to improve quality. The primary synthesis method of CNTs was based on carbon arc discharging, but other procedures such as carbon laser ablation and chemical vapor deposition (CVD) were also used for making CNTs [177].

Early studies on producing CNTs were based on carbon arc-discharge, but in this section, various synthesis techniques for both CNTs are presented. In 1991, Ijima produced CNTs using an arc discharging evaporation method like fullerene production. In this procedure, CNTs were grown on a negative carbon electrode cathode in an Ar chamber by passing direct current and making the arc discharge between the electrodes. In order to have high quality of produced CNTs, it needs to eliminate any defects over a significant length scale (such as 1–10 μm) along the tube axes. There are many challenges in producing high-quality CNTs, such as having a large scale and low cost synthesis method that provides controllable design conditions to have the desired electrical properties, orientation and length of tubes [10].

Various techniques have been used for producing CNTs with different structures and properties [12]. The first massive production of CNTs was reported by Ebbesn and Ajayan [178,179]. They used the same method that Ijima applied for CNT synthesis and their studies opened new doors to the subsequent efforts on massive production of CNTs. There are three main approaches to produce CNTs: arc-discharge method, laser ablation and thermal synthesis.

#### 5.1.1. Arc-Discharge

Arc-discharge was the first approach for obtaining single-walled and multi-walled carbon nanotubes [4]. The arc-discharge procedure happens in a low voltage (~12–25 V) and high current (50–120 A) arc welder. The arc is produced between two graphite electrodes. The distance between them is about 1 mm. The electrodes are placed in inert gas (helium or argon) at a range of 100–1000 Torrs of pressure. Carbon nanotubes are formed on the cathode along with other byproducts. This method was used for the first time for producing MWCNTs. Literally, Ijima and Bethune found that utilizing catalysts can lead to forming only SWCNTs [11]. They used iron or cobalt metals on the anode as catalysts [180].

The high-temperature discharge is provided by applying a current up to 200 A. The carbon on the surface of the electrodes is vaporized and forms CNTs on the other electrode surface. The multi-component arc discharge condition was reported as a critical point for the synthesis of the CNTs. Several researchers have reported the production of CNTs based on arc discharging methods [181,182,183].

#### 5.1.2. Laser Ablation

**Figure 22:**
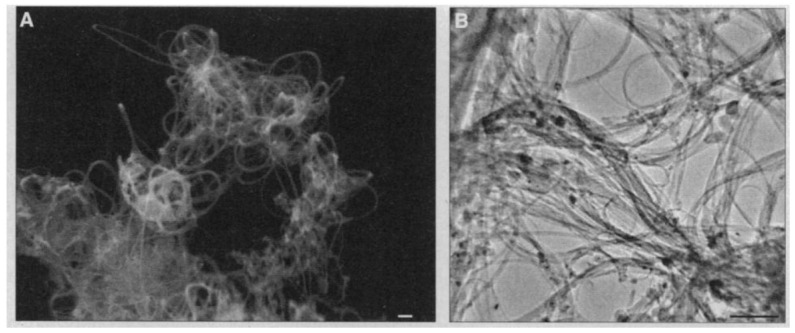
(**A**) An SEM image of long SWCNT carbon fibers with diameters ranging from 10 to 20 nm (scale bar 100 nm). (**B**) A low-resolution image including all of the SWCNT ropes and catalyst particles coated with amorphous carbon (scale bar 100 nm) [184].

Laser ablation provides an efficient synthesis of narrow diameters of SWCNTs. The first report of this method was based on high yield (>70–90%) conversion of graphite to nanotubes [184]. Two laser pulses from a Co/Ni graphite composite laser were employed to evaporate the carbon/transition metal target (Figure 22) in the presence of Ar atmosphere and high temperature. The tubes which were produced in this study are 10–20 nm in diameter and up to 10 μm in length. In the same year, the Smalley group published an article about large-scale production of SWCNTs with the laser ablation method. In their method, the resulting CNTs and other byproducts were collected on the other front of the inlet in the tube furnace. The arc-discharge and the laser ablation approaches are similar in principle but differ from each other based on the metal used to impregnate the graphite for producing CNTs. Powder samples and twisted tubes can be synthesized with these two methods. As these methods require a vacuum and high temperature and continuous target replacement, they are considered as complex methods for producing a large number of CNTs.

#### 5.1.3. CVD

Another efficient technique to produce CNTs in a solid-state is CVD. The main idea behind is a gaseous hydrocarbon decomposition with metallic nanoparticles with nucleation roles in the growth of CNTs. This method has been used as a preferred procedure for the scaled production of CNTs [45,185]. CNTs have been profitable nanostructures for more than 20 years in producing various carbon structures such as fibers, filaments and tubes [186,187]. In the CVD method, high temperature is applied to a catalyst (50–1000 °C). Hydrocarbon flows in the tube and the catalyst forms nanoparticles on the surfaces of alumina substrates. This mechanism of growing involves hydrocarbon decomposition when metallic catalysts are present. Carbons dissolve in the metal nanoparticles and subsequent deposition of carbon occurs in the tubular *sp*^2^ form.

Ethylene or acetylene are usually used as gaseous hydrocarbon and iron, nickel or cobalt play the metal transition catalyst role during the process. The CVD method is generally considered when producing large-scale CNTs with the controllable direction of tubes is required [188]. The main advantage of this method is that the tubes with well-organized structures can be obtained at lower temperatures.

**Figure 23:**
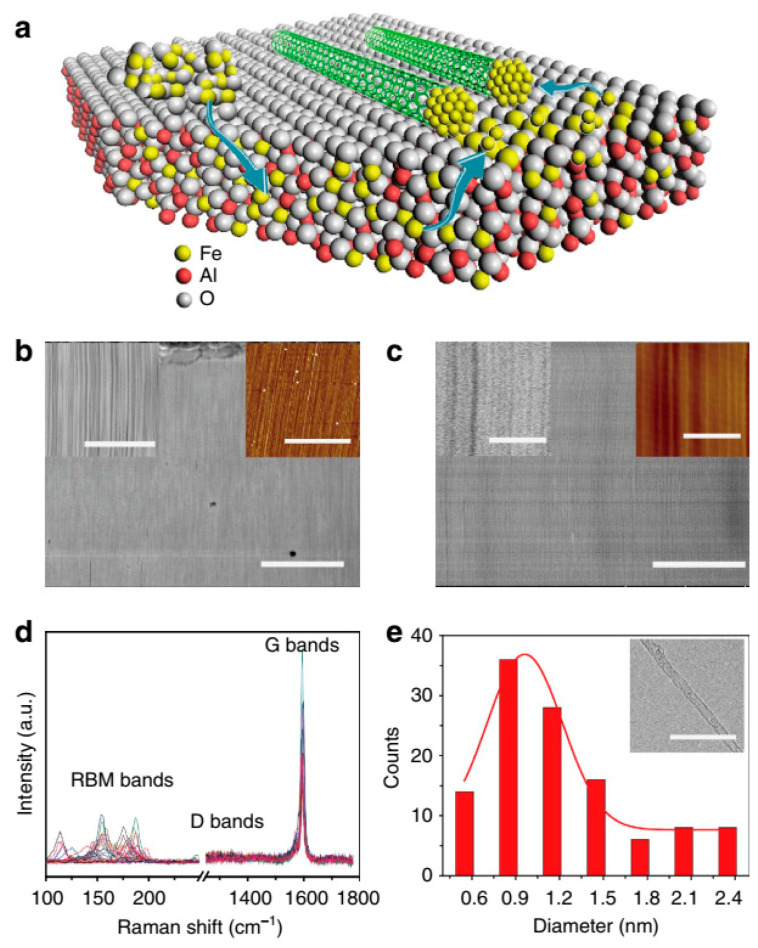
Schematic illustration of the growth mechanism and characterization of high-density SWNT arrays. (**a**) Schematic illustration of the growing process. (**b,c**) SEM and AFM images under different magnification. (**d**) Raman spectra with 514.5 nm excitation. (**e**) Diameter distribution. The red solid lines are Gaussian fitting peaks. Scale bar, 50 mm (**b**) (scale bar, 3 mm for the insets in b). Scale bar, 300 nm (**c**) (scale bar, 50 nm for the insets in c). Scale bar, 10 nm (**e**) [189].

With the CVD method, high density and quality of SWNT arrays can be produced, which is vital to realize the SWNT-based IC applications. The catalyst is a key factor in this method. How to maintain the activity of catalysts, give catalyst nanoparticles more opportunities to nucleate SWNTs and provide new catalysts during growth are key issues for obtaining SWNT arrays with ultra-high density on substrates. Hu proposed an approach to grow SWNT arrays with ultra-high density using Trojan catalysts [189]. Figure 23a schematically illustrates the procedure of this method. The idea behind this method is firstly storing the catalysts by dissolving into the substrate and then gradually releasing under hydrogen atmosphere during the growing process. This gradual release mode helps reduce the interaction between active catalysts. The SEM and AFM images in Figure 23b,c prove the high density of at least 130 SWNTs μm^−1^. Figure 23d,e indicates the high quality of the obtained SWNT arrays.

**Figure 24:**
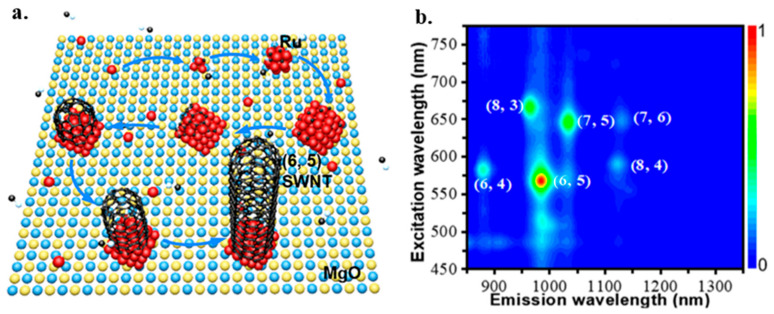
(**a**) Schematic illustration of Ru particle formation from AD-Ru/MgO for catalyzing the nucleation and growth of an SWNT. (**b**) Contour plot of normalized PL intensities under the various excitation energies [190].

A proper catalyst can also be used to realize the chirality-selective synthesis of SWNTs in the CVD growth method. Zhang demonstrated the chirality-selective synthesis of SWNTs with the monometallic AD-Ru/MgO catalyst at 850 °C in CVD [190]. Figure 24a illustrates the catalyst activation mechanisms. During the growth process, highly dispersed Ru atoms in AD-Ru/MgO aggregate and form Ru clusters suitable for the nucleation and growth of sub-nanometer SWNTs with dominant (6, 5) tubes. The activation of Ru catalysts supported on MgO is attributed to the charge transfer from substrate to metal clusters, promoting the carbon source decomposition and SWNT cap stabilization. PL spectroscopy characterization results in Figure 24b show that the preferential synthesis of (6, 5) SWNTs was obtained.

#### 5.1.4. Other Methods

Recently, other methods, such as vapor phase growth (VPG), flame synthesis, and nebulized spray pyrolysis, have been studied to produce CNTs [191,192]. VPG is similar to CVD, but carbonous gas and catalyst are placed in the chamber without a substrate in the VPG method. In the flame synthesis method, a flame consisting of hydrocarbon is used to grow CNTs, assisting the catalyst particles. The growth occurs in the same manner as in CVD [193,194].

Another method is nebulized spray pyrolysis, in which a unique ultrasonic atomizer has been employed. The catalyst is required here, and ethanol is sprayed into the furnace at a high temperature of 800 °C. This method with lots of benefits has been considered by many researchers [195,196].

Table 3 provides a summary and comparison of the primary synthesis methods of CNTs.

### 5.2. Growth Mechanisms

This is an appropriate point to mention that all the excellent properties quoted for CNTs describe an atomically ideal CNT structure. However, depending on the synthesis method used for the production process of the CNT, the result could be very different from theoretical expectations. Although many studies have been undertaken to build high-quality CNT structures, because of a lack of complete understanding of mechanisms and a well-established model [197], researchers have not been able to construct an effective method to produce CNTs with near-ideal properties for large-scale production.

When hydrocarbon vapor encounters hot metal nanoparticles, it will decompose into carbon and other hydrogen species. After the scattering of the hydrogen, carbon would be dissolved into metal. After reaching saturation, the dissolved carbon will precipitate and cause an energetically stable cylindrical crystallization with no dangling bonds. A thermal gradient will cause the process to continue, which will be outlined as follows. The decomposition of the hydrocarbon delivers some heat to the exposed area of the metal. On the other hand, the crystallization of the carbon as the endothermic procedure absorbs some heat from the precipitation regions of the metal.

**Figure 25:**
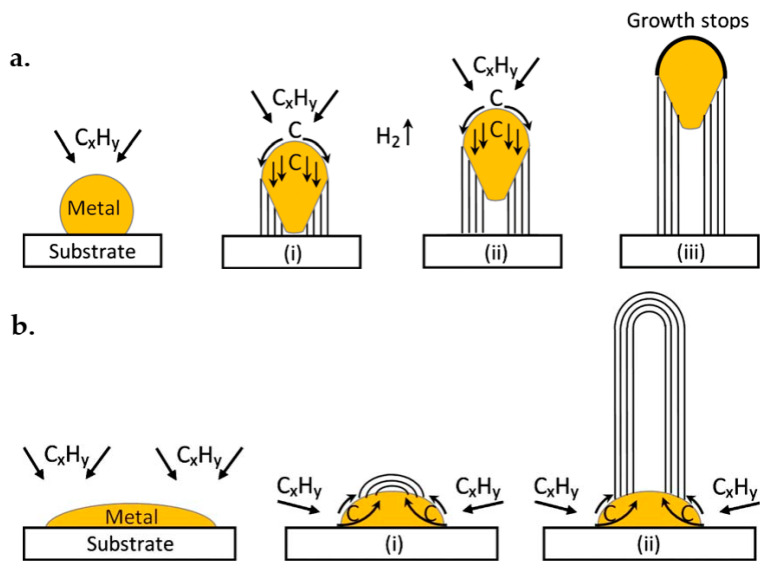
Well-known established growing method of CNTs. (**a**) Tip-growth illustration and (**b**) base-growth illustration [197].

As depicted in Figure 25, two well-accepted models for growth mechanisms are the tip-growth model and the base-growth model. Each of these mechanisms will form depending on the strength of the interactions between the substrate and the catalyst, which is actually the contact angle between the metal and the substrate (acute or obtuse, respectively) [197,198,199].

#### 5.2.1. Tip-Growth Model

In this model, the substrate–catalyst interactions are weak, see Figure 25a. The contact angle between metal and substrate is acute. First, hydrocarbons are decomposed on the metal surface. After carbon diffusion through the metal, CNT would precipitate on the bottom of the metal and push the metal particle off the substrate. The concentration gradient keeps permitting the carbon to diffuse, and the CNT keeps growing in length. The process will happen because the top of the metal can receive hydrocarbon decomposition. The CNT growth is stopped when the activity of the catalytic is done, and the metal is completely covered with carbon.

#### 5.2.2. Base-Growth Model

In the base-growth model, as depicted in Figure 25b, the metal–substrate contact angle is obtuse, and substrate–catalyst interaction is strong. This model also involves hydrocarbon decomposition and carbon diffusion, similar to the tip-growth model. Still, the catalyst particle is rooted in the base on the substrate, and the CNT is growing up upon it. However, the interaction with the substrate is minimum, and the precipitation will come out from the apex of the metal. Furthermore, carbon crystallizes in the semispherical shape and is extended towards a graphitic and ideal cylindrical form. The upcoming hydrocarbon decompositions occur in the lower outlying layer of the metal surface, and the dissolved carbon diffuses upward.

**Figure 26:**
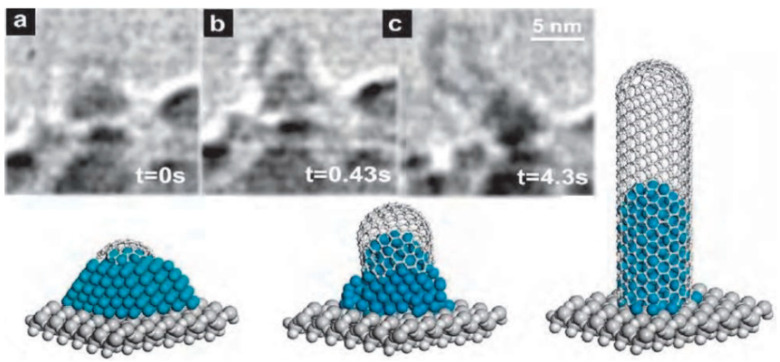
ETEM image of Ni-catalyzed CNT root growth at different time, (**a**) 0 s, (**b**) 0.43 s, (**c**) 4.3 s [183].

Although these are the general models for the growth mechanism, one cannot state the physical state of the catalyst and if the metal is in a solid or liquid phase during the growth mechanism. Furthermore, one cannot know if carbon diffuses in the surface diffusion mode or the volume diffusion mode. Additionally, whether the catalyst should be metal carbide or pure metal is yet to be determined. Several experiments reported such aspects of the growth mechanism [183,200,201,202,203,204,205,206,207,208,209,210]. Figure 26 shows an in situ sequence of TEM images on the growth mechanism for SWCNT. In this experiment, a small Ni cluster led to the formation of an SWCNT via the base-growth mechanism at the temperature of 888 K. Researchers reported that the carbon cap appeared and had a smaller diameter than the Ni cluster. Moreover, the cluster’s peak took the form of a cylinder and eliminated the carbon cap from the cluster and caused the formation of an SWCNT [183].

## 6. Characterizations

Many techniques have been used to identify graphene materials. The most commonly used methods are AFM, transmission electron microscopy (TEM), X-ray diffraction (XRD), Rayleigh spectroscopy and Raman spectroscopy. Moreover, energy-efficient electronic systems have been proposed by combining machine learning to those traditional tools, which help to meet the requirement in the field of nanoelectronics’ research, development and manufacturing.

### 6.1. AFM

**Figure 27:**
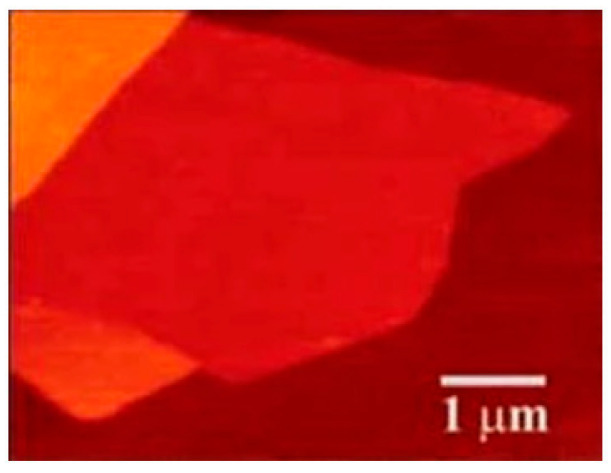
AFM image of graphene with different thicknesses. The dark brown region is SiO_2_ substrate. The brownish red, yellowish brown and orange regions are graphene layers with a thickness of 0.8, 1.2 and 2.5 nm, respectively [124].

The AFM technique is widely used in testing graphene layer thickness. This is often done in the tapping mode. It should be noted that the thicknesses of 2D films may be raised by a few angstroms because of the water absorption between layers. Hence, the overlaid layers deserve detailed investigation when testing one layer thickness. However, this method’s efficiency is low. To make it worse, the measured results by different groups sometimes are even controversial [124]. Both Novoselov and Gupta tested the thickness of single-layer graphene using the AFM method but got quite different results. The former measured a thickness of 0.4 nm, while the latter got 0.7 nm. Figure 27 displays graphene’s AFM image [124].

The inconsistency might be due to the selection of cantilevers with different free amplitude in their experiments. Although the AFM method has been widely used in graphene thickness measurements, the inconsistent results show that it may be not reliable and deserves further investigations [211].

### 6.2. TEM

**Figure 28:**
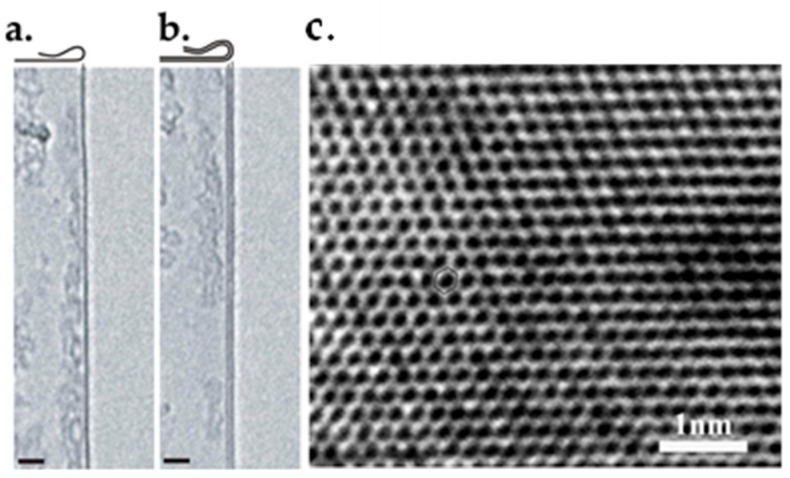
TEM images of folded graphene edge, (**a**) single layer and (**b**) bilayer [212]. (**c**) HRTEM result of single-layer graphene [170].

TEM is the most accurate approach to characterize graphene layers. It can be used to detect both single- and multilayer graphene. Especially, this technique has been frequently used in detecting folded graphene sheets, which are parallel to the electron beam. As illustrated in Figure 28a,b, the folded edge of single-layer graphene is visualized by one dark line, which would be two dark lines in the case of bilayer graphene [212]. However, caution must be taken when analyzing TEM results because the scrolls, the multiple overlayers and even single-layer graphene would be visualized as several dark lines. Figure 28c demonstrates an HRTEM image of single-layer graphene [170].

The TEM technique is of high accuracy, but it is time-consuming. Moreover, additional techniques are needed to prepare the samples. Therefore, it is a good option when undertaking fundamental research on graphene [213].

### 6.3. Raman Spectroscopy

**Figure 29:**
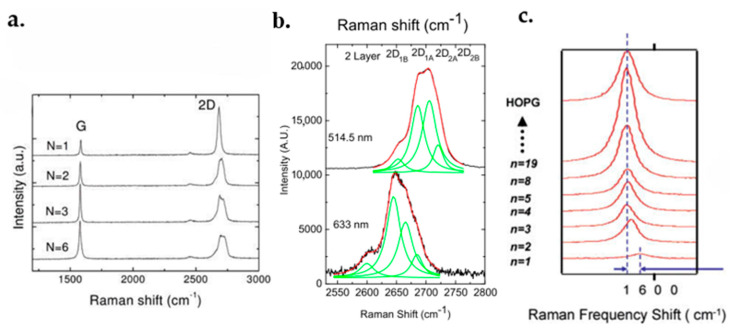
(**a**) Raman results of graphene with different layer thicknesses [213]. (**b**) Bilayer graphene 2D band transformation at 514 and 633 nm [214]. (**c**) The G band is due to the different layer numbers [215].

Raman spectroscopy is an analytical technique where information of inelastically scattered photons is collected to characterize graphene. It is thought to be the most suitable method for graphene characterization due to its accuracy and the ability in identifying the layer numbers, the doping level and the quality of the flakes. Figure 29a demonstrates the Raman results of graphene with different layer thicknesses [213].

It should be noted that three bands, namely the D band, the G band and the 2D band, have to be considered when analyzing the graphene layers’ Raman data. The Raman spectra of suspended and on-substrate graphene are usually similar. The main difference could be a small D peak in the TEM samples [214]. This difference could be the result of a coincidence between the Raman bands of the substrate with bands of graphene making subtraction of the substrate bands necessary.

The G band location shows the graphene layer numbers. As shown in Figure 29c, the G band peak of the single-layer graphene is at about 1587 cm^−1^, and shifts left with increasing layer numbers. The G bands of graphene with various amounts of thickness are displayed in [215].

The D band is recognized as a defects band, which illustrates defects and carbon *sp*^3^ bonds in graphene layers. High-quality graphene may have a low-intensity D band. This peak can be detected at the graphene flake edges [216].

The 2D band is the second order of the D band being always very strong in graphene. The peak shape gives the information of layer numbers. A single layer graphene has a symmetrical 2D band peak with a narrow full width half maximum (FWHM). As for multilayers, the peak changes into a waveform containing multiple peaks. Figure 29b demonstrates a waveform of double-layer graphene [215,216].

**Figure 30:**
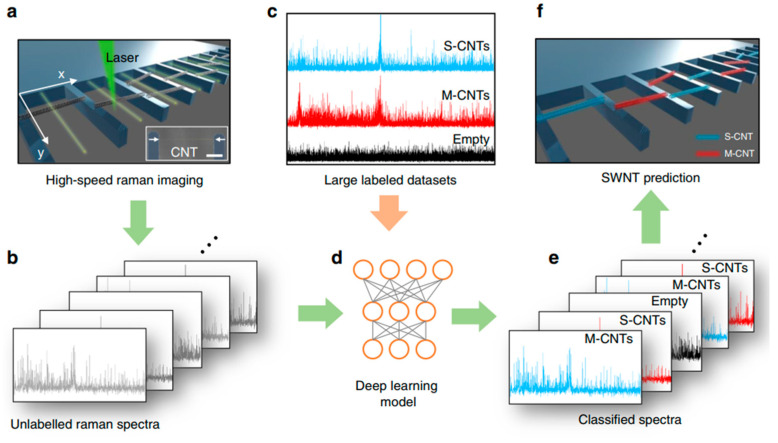
Schematic illustration of CNT characterization using deep-learning-based Raman spectra analysis. (**a**) Implementation of high-speed Raman imaging on a fork-like growth substrate. (**b**) Generation of unlabeled Raman spectra. (**c**) Large labeled datasets organized into three classes: S-CNTs, MCNTs and empty. (**d**) Deep learning model. (**e**) Classification of individual spectra using the model. (**f**) CNT identification [217].

Figure 30 schematically illustrates the suspended CNT characterization using deep learning-based Raman spectra analysis. By using a Raman spectroscopy line-scan method and deep learning classification method, the Raman scanning rates were significantly increased. Moreover, laser-induced sample damage was minimized by reducing the exposure time to only a few milliseconds. This method permits the quantitative identification of CNTs on the growth substrate, providing information about their number, position and physical properties [217].

### 6.4. Rayleigh Spectroscopy

Rayleigh spectroscopy is also used for the characterization of graphene. Compared to Raman spectroscopy, the Rayleigh method is also a fast, non-destructive and accurate method, though it is based on elastic scattering. It has been used for identifying monolayer and multilayer samples. In this method, the difference in intensity between the substrate and sample can be identified by an image contrast, when the substrate intensity is normalized.

**Figure 31:**
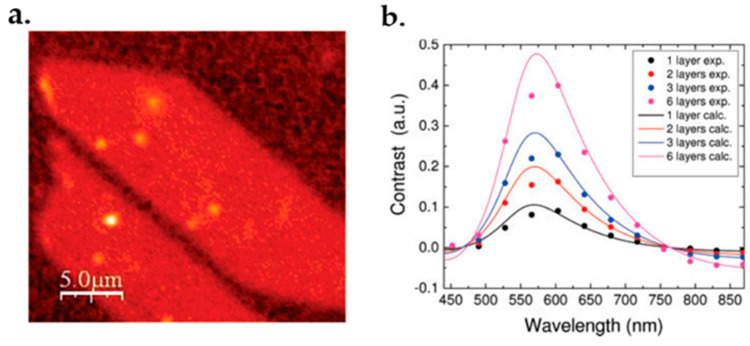
(**a**) Confocal Rayleigh result of monolayer graphene obtained by raster scanning the test subject; (**b**) the differences between results gained by calculation and empirically with regard to wavelength for a distinct amount of layers [213].

An interferometrically detected signal of graphene films was obtained using Rayleigh scattering by Casiraghi et al. In his research, the background intensity was treated as a reference beam for the better detection. The result is shown in Figure 31a [213].

About 0.08 contrast at 633 nm wavelength was detected for monolayer graphene. Other wavelengths were also used for characterization and the contrast for different graphene layers was found. Figure 31b demonstrates the Rayleigh contrast for different graphene layers measured at different wavelengths [213].

### 6.5. XRD

**Figure 32:**
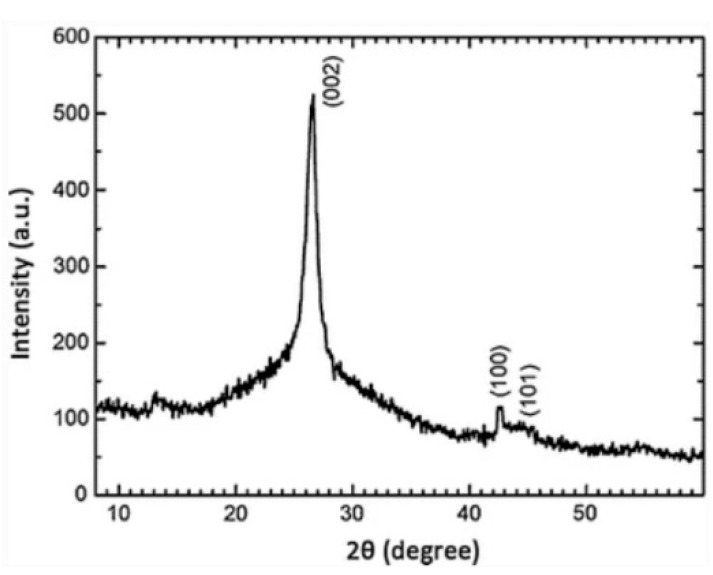
XRD pattern of graphene nanosheets [218].

Another technique for graphene characterization is XRD. Three peaks could be detected when testing the graphene materials using XRD. A strong peak at 26.3° stands for the 002 plane. The peaks at 43.2° and 44.6° represent 100 and 101 planes, respectively, as shown in Figure 32 [218]. The 002 peak indicates the thickness of the graphene film.

**Figure 33:**
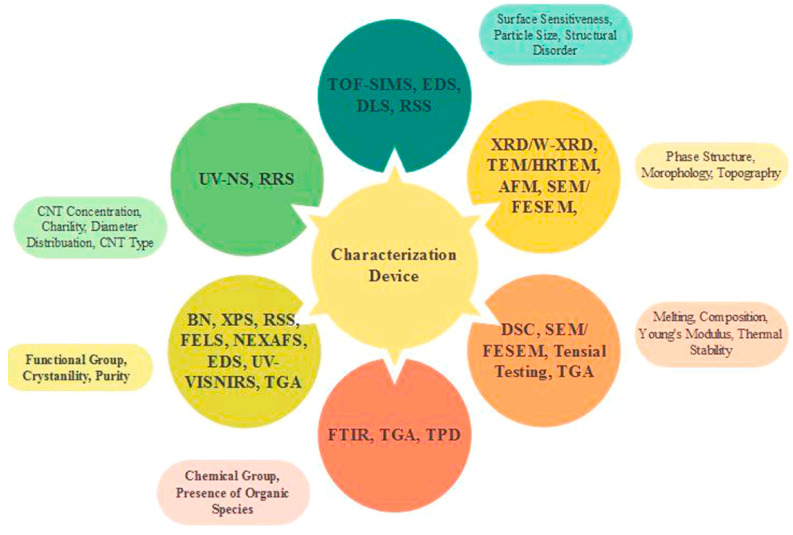
Schematic of the graphene characterization methods and the related devices [219].

Figure 33 schematically displays several characterization approaches for graphene materials. For graphene’s morphology characterization, electromagnetic spectroscopy, nuclear magnetic resonance (NMR), XRD, AFM, electron microscopy and light scattering methods are most commonly used. As for the confirmation of graphene’s functional groups on graphene, the Raman spectroscopy, NMR and IR spectroscopy are utilized [219].

## 7. Graphene Applications

In this section, applications of graphene in micro- and nanoelectronics, optoelectronics and biotechnology will be introduced.

### 7.1. Graphene FET

MOSFETs’ down-scaling may be the most effective way to achieve high-performance low-power targets in CMOS technology. However, further down-scaling may cause a few behaviors, such as velocity saturation, current leakage, mobility degradation, velocity saturation, narrow width and short channel effects [220]. Hence, researchers are seeking new materials to substitute silicon-based devices. Graphene is considered to be one of the options due to its extraordinary electronic features, such as high current density, ballistic transport and long electron mean-free-path. The International Technology Roadmap for Semiconductors (ITRS) considers graphene as one of the candidate materials for post-silicon devices [221]. It should be noted that graphene was initially applied in the FETs [28]. FETs are utilized in two important applications, which are logics similar to CMOS and radio frequencies (RF). For logic applications, an FET should be able to turn on and off with large on/off current ratios in the range of 10^4^–10^7^, corresponding to the channel’s bandgap of approximately 0.4 eV [221]. Moreover, symmetric threshold voltages are needed for both the n-type and p-type FETs. However, for RF applications, on-state is always needed and no switch-off operation is required. Graphene is usually utilized in this field, as will be discussed later.

**Figure 34:**
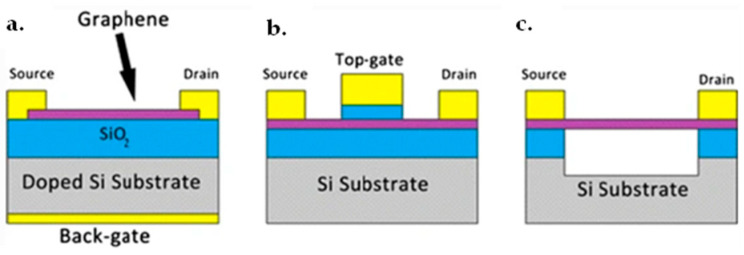
Three types of graphene FETs: (**a**) back-gated, (**b**) top-gated and (**c**) suspended.

Many studies have been undertaken on graphene FETs (GFETs), which use graphene as the channel [28,90,222,223,224,225,226]. Figure 34 displays different kinds of GFETs. We can see a device’s evolution trend if looking back from the very first GFET. Back-gate devices were first introduced. The back-gate electrode and dielectric were made using doped Si and SiO_2_, respectively, as shown in Figure 34a. However, this design is not suitable as GFETs. The second generation was fabricated with top-gate electrodes, see Figure 34b [227]. It is also reported that a hot-electron transistor using a base contact graphene demonstrated remarkable DC and RF performance [228]. However, the mobility of the expected graphene channel would be degraded during the deposition of the dielectric and gate material deposition on the graphene layer [229]. The third generation, called suspended graphene, was recommended in 2007 to solve the challenges, see Figure 34c. Double gate GFETs are also introduced with back-gate and top-gate. Despite the structure of such devices, another challenge is the large contact resistance caused by the non-optimum alignment of the gate, source and drain. This may result in device malfunctions.

Because the single-layer graphene is a semi-metal with zero bandgap at room temperature, GFETs cannot be turned off and have low on/off current ratios. To overcome this issue, many methods were proposed to introduce a bandgap into graphene, such as producing graphene nanoribbons (GNRs) [88], biasing double-layer graphene [90], strain application [230] and doping [231]. The outcomes of these approaches on FETs are discussed below.

GNRs have large on/off current ratios above 10^5^, which are suitable for logic applications [225,232]. If a CNT can be treated as rolled-up graphene flakes, then a GNR is simply an unrolled CNT. GNRs and CNTs have similar electronic properties. The properties of SWCNT depend on the tube chirality and diameters, while the GNR’s properties depend on the ribbon widths and directions. Nevertheless, the sub-10 nm GNRs have an all-semiconducting feature, which could solve the issues in CNTs whose metal/semiconductor feature extremely depends on the chirality [128]. Smooth edges and narrow widths are mandatory to make a semiconducting GNR. Thus, it is challenging to produce massive GNRs with controllable edges and widths. Nevertheless, a tiny disorder at the GNR edge would introduce a bandgap [233].

Since the properties are greatly structure dependent, it is essential to synthesize atomically precise GNRs to fulfill the targeted function. A number of top-down approaches have been developed for the fabrication of GNRs, such as lithographic [88,234,235], chemical [236], sonochemical [232] and plasma etching [139]. However, these approaches can hardly control the GNRs’ width and edge structure at the atomic level. Thus, the obtained chemically less defined GNRs lack accurate control over the properties. On the other hand, beginning with the tailor-made organic precursors, atomically precise GNRs can be synthesized based on bottom-up chemical synthesis approaches.

The bottom-up approach mainly consists of two main pathways: solution-mediated synthesis and surface-assisted synthesis [237]. The former corresponding polyphenylene precursors are constructed such that the desired GNRs can be obtained by C-C bond formation between the benzene rings. This solution synthesis can be scaled up to the gram scale [238] and allows various edge functionalization. It should be mentioned that the introduction of long alkyl chains and other bulky functional groups render the resulting GNRs dispersible in organic solvents, which allow for the liquid-phase processing for further characterizations and device fabrication. In the case of surface-assisted synthesis, homolytic carbonhalogen cleavage is thermally induced, and the obtained diradicals undergo polymerization to form linear polymers on a metal surface, such as Au (111). Subsequent annealing at higher temperatures results in the formation of GNRs through surface-assisted intramolecular cyclodehydrogenation. It was initially carried out under ultrahigh vacuum (UHV) conditions with pressures lower than 10^−9^ mbar. Recent progress moved to use an industry-viable CVD setup under less-demanding high vacuum (HV), lower vacuum and even ambient pressure conditions. GNRs of same structures have been obtained comparing to those obtained under UHV. Although the length of the GNRs and the defect density might be compromised, the CVD method can increase GNR production and reduce costs, which are critical requirements for wider application.

It is reported that transistors with double-layer graphene have on/off current ratios of about 100 and 2000 at room temperature and 20 K, respectively [90]. It is good for the high mobility application. However, it is still not enough for logic applications.

Monolayer graphene appears to be better in RF applications compared to logic applications because the transistors are not required to be turned off. Because the cut-off frequency increases with mobility, large area graphene layer could be used directly as the transistor’s channel [239]. These RF devices have high cut-off frequencies, owing to the graphene channel’s high mobility. However, low current saturation may degrade the power gains, cut-off frequencies and *f_max_*.

**Figure 35:**
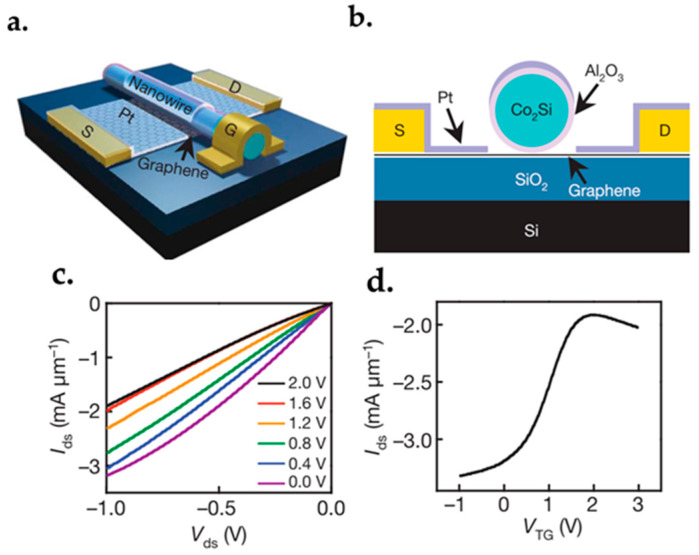
The 300 GHz RF GFET: (**a**) 3D view, (**b**) front view, (**c**) the *I_ds_-V_ds_* curves at different gate voltages and (**d**) the *I_ds_-V_TG_* curve at *V_ds_* = −1 V [241].

RF devices are usually utilized in military applications. In these devices, one of the most important parameters is the cut-off frequency (the maximum frequency where the device works properly). It has been reported that RF GFETs can operate at about 100 [222] and 155 GHz [240] cut-off frequencies. As yet, the fastest RF GFETs (144 nm gate length) with a cut-off frequency of 300 GHz have been reported [241]. It is two times faster than the best silicon FETs with similar sizes and comparable to the fastest III-V HEMTs with similar gate lengths. As shown in Figure 35a,b, the top-gate of the transistor is a self-aligned C_o2_Si-Al_2_O_3_ core-shell nanowire, below which the graphene layer is located on a Pt substrate. The nanowire’s diameter is defined as the transistor’s gate length. The insulate Al_2_O_3_ shell works as the dielectric, the metallic C_o2_Si core works as the top gate, and the thin-Pt-film pads work as the transistor’s drain and source. Figure 35c demonstrates the *I_ds_-V_ds_* characteristics of the FET at different top-gate voltages (*V_TG_*). Figure 35d displays that the device has a maximum scaled 3.32 mA/μm on-current at *V_ds_* = −1 V and *V_TG_* = −1 V.

It was discovered that the increasing charged impurities can decrease graphene mobility. Additionally, surface functionalization may break the chemical bonds in graphene lattice or introduce unwanted impurities. Consequently, SiO_2_ was substituted with high- κ materials, such as HfO_2_, to decrease the density of the unwanted impurities [242]. The high-κ dielectrics can reduce the impurity scatterings due to the increased screening effect [243].

### 7.2. Light-Emitting Device

**Figure 36:**
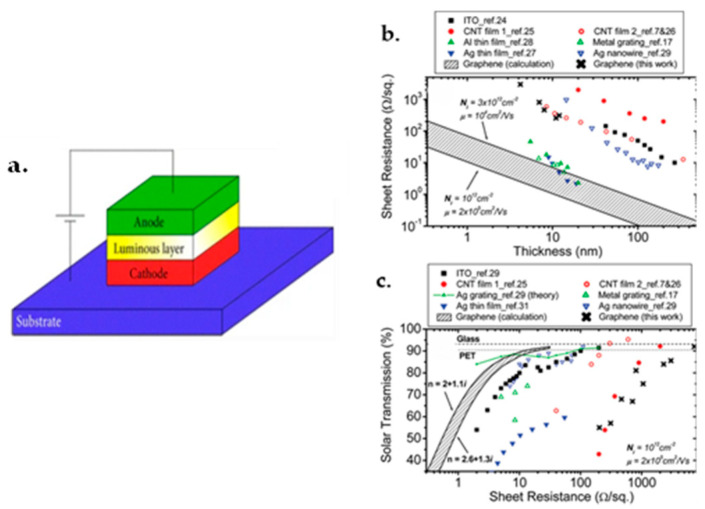
(**a**) Typical structure of an OLED. (**b**) Sheet resistance decreases with the increase in thickness. (**c**) The solar transmission increase monotonically as a function of sheet resistance [244].

Organic light-emitting diodes (OLEDs) have many advantages over traditional LEDs, such as high luminous efficiency, cost-effective fabrication and compatibility with different substrates. The invention of OLEDs is considered to be a big scientific success because not everyone expected carbon-based organic materials to emit light. The typical structure of an OLED is schematically illustrated in Figure 36a. It contains a cathode, an anode and an organic layer. The layer is often made of polymer materials such as phenyl or naphthalene-1-yl and can emit light in response to an electric current. When an electric current passes the diode, electrons from the cathode are injected into the layer’s LUMO, and holes from the anode are injected into the layer’s HOMO. The electrons and holes recombine in the layer, creating excitons, which would decay and emit light.

The electrode materials of the OLEDs shall meet some requirements. The cathode is required to have a low resistance and low work function (WF). Nevertheless, the anode is required to have a low resistance but high WF [245,246]. Anode transparency is vital to the performance of the OLEDs [247]. Indium tin oxide (ITO) (4.4 ≤ WF ≤ 4.5 eV, resistivity of 2–4 × 10^−4^ Ωcm^−1^) is often used as transparent anode materials [248,249]. However, it has drawbacks. First, the price could be high because of the diminishing of In resources on earth [245]. Second, the In element would defuse in the polymer layer, which may degrade the device performance [250]. To solve that issue, graphene was selected as an alternative material for ITO. The graphene sheet has a high WF (4.2–4.6 eV). Moreover, it has a low resistance of <10 Ωcm^−1^ when the thickness is below 10 nm, whereas the minimum thickness of ITO is 100 nm to obtain a similar resistance [251]. As previously described, the graphene sheet resistance and transmission decrease with the increasing thickness. Consequently, a trade-off exists between the transmission and the resistance. The graphene sheet resistance follows:

(9) $$R_{sh}=\frac{1}{e\mu N_{i}N}=\frac{62.4\Omega }{N}$$

where *N* is the number of single-layer graphene.

Figure 36b displays the sheet resistance ($\frac{\Omega }{sq.})$ as a function of the thickness (nm). Figure 36c shows the solar transmission (%) as a function of sheet resistance ($\frac{\Omega }{sq.})$ [244]. These two diagrams illustrate the tradeoff effect.

**Figure 37:**
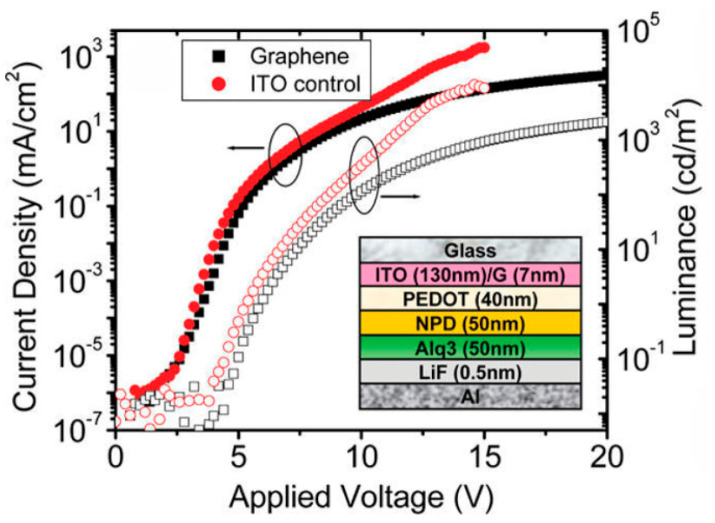
The current density (solid symbols) and the luminance (open symbols) as a function of applied forward bias voltage for an ITO (circles) and graphene OLED (squares) [244].

An OLED fabricated using a 7 nm-thick graphene film as the transparent anode has been reported by Wu et al. [244]. The graphene was made by a thermally reduced method and has a sheet resistance of ~800 ($\frac{\Omega }{sq.})$ and a transparency of 82%. In another group, Kim et al. achieved a sheet resistance of about 280 ($\frac{\Omega }{sq.})$ and a transparency of 80% [153]. Figure 37 displays the current density and the luminance of ITO and graphene as a function of applied voltage. Graphene device has a comparable luminance below 10 cd/m^2^ and a comparable current density below 10 mA/cm^2^ with the ITO device. For a 300 cd/m^2^ luminance, the *V_t_* of the graphene OLED and the ITO OLED are 11.7 and 9.9 V, respectively [244].

**Figure 38:**
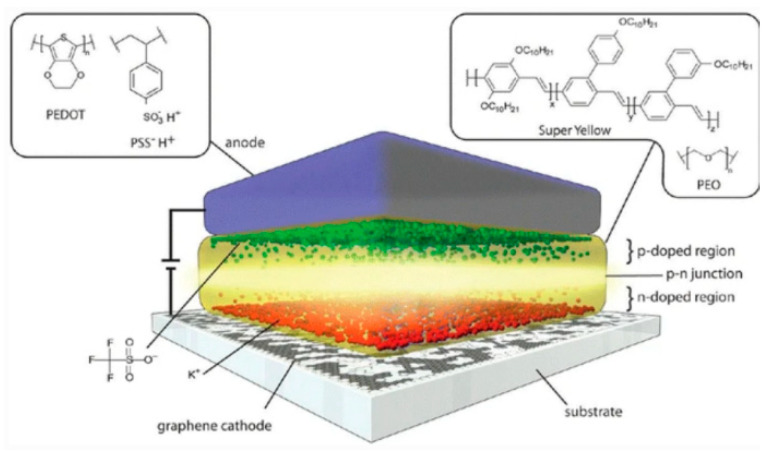
Body of an LEC. The chemical formulas and the formula icons of every layer are also displayed [246].

Since graphene has relatively high WF, it is unreasonable to consider graphene as a good cathode. However, many groups use it as the cathode in a new type of device called a light-emitting electrochemical cell (LEC) [246]. The structure is similar between LEC and OLED. The only difference is that the luminous layer in LEC is an electrolyte. Therefore, there is no need to match the WFs. The ions in the electrolyte will redistribute and form a p-i-n junction when an electric field is applied between the cathode and the anode [252]. Figure 38 schematically demonstrates an organic LEC structure [246]. The cathode was made of chemically derived graphene (CDG) and the anode was made of the poly 3, 4-ethylene dioxythiophene-poly styrene sulfonate (PEDOT-PSS). The active layer was a mixture of poly paraphenylene vinylene copolymer (or “Super Yellow” (SY)) and a dissolution of KCF_3_SO_3_ salt in poly ethylene oxide (PEO).

**Figure 39:**
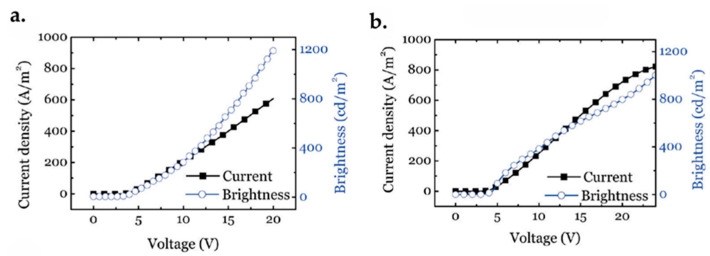
Brightness (circles) and current density (squares) as a function of applied voltage (**a**) measured through a graphene cathode and (**b**) measured through a PEDOT-PSS anode [246].

Lights could couple out of both sides in this device, because the electrodes were transparent in this research. Figure 39 demonstrates the brightness and current density as functions of applied voltage measured with a graphene cathode and PEDOT-PSS anode, respectively. Matyba et al. also reported an LEC device using graphene as the anode [246]. All these studies indicate that graphene is a promising material in the light-emitting devices market.

### 7.3. Energy Storage/Conversion Devices

**Figure 40:**
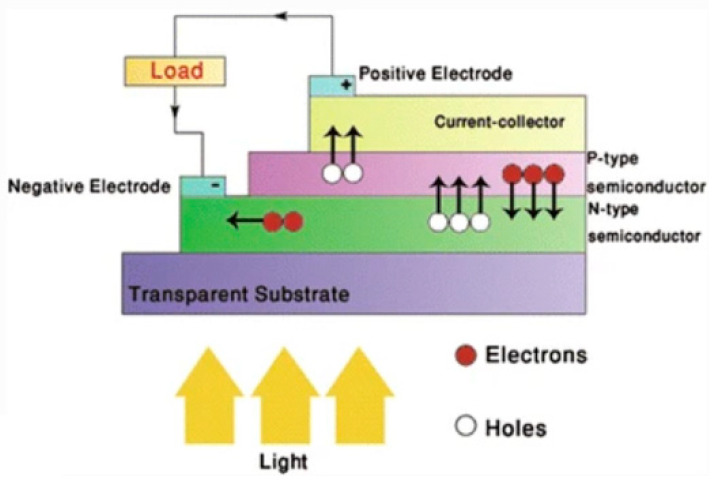
Schematic diagram of a typical solar cell structure.

Heteroatom-doped and co-doped graphene-based materials (n-type and p-type doping) have been synthesized for devices in energy-related devices such as solar cells [253,254], batteries, fuel cells, water splitting and supercapacitors. Figure 40 demonstrates a typical solar cell structure. It has been reported that graphene can be used in solar cells in three different ways [129,255,256,257], which will be discussed below.

#### 7.3.1. As the Window Electrode

The transparent window electrode (conductive electrode), where light couples in or out of the devices, is a figure of merit in optoelectronic devices. For the traditional solar cells, ITO is often utilized as the window electrode material. However, graphene has advantages over ITO as explained above. Wang et al. have reported a window electrode using a 1000–3000 nm-thick thermal reduced graphene sheet with a high conductivity of 550 S/cm and transparency of 70% [129]. Recently, Wu et al. utilized a solution-processed 4–7 nm-thick graphene film as the conductive anode in the organic photovoltaic cell. The film has a resistance of 100–500 kΩ/sq and a transparency of 85–95% [256]. It should be noted that the solution-processed graphene sheets may contain many lattice defects and oxidative traps. Therefore, to get proper sheet resistances, the graphene sheet number shall be increased. Because the transparency and resistance decrease with increasing film thickness, this tradeoff shall be taken into consideration before making a viable conductive electrode. Compared to that of ITO-based solar cells, the performance of graphene-based solar cells is decreased because of the high sheet resistance of graphene films [256].

#### 7.3.2. As an Acceptor

**Figure 41:**
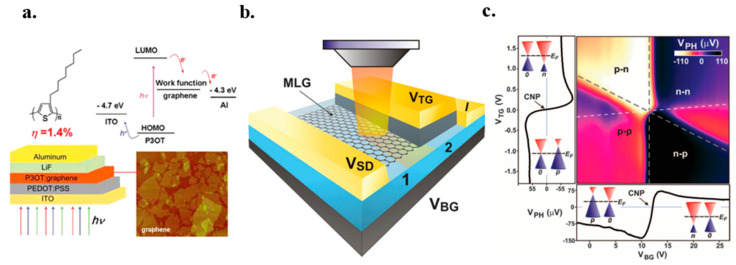
(**a**) A graphene solar cell structure where the acceptor layer is made of graphene sheet [255]. (**b**) A schematic diagram of a graphene solar cell showing the laser excitation, top gate and back gate and the graphene layer [257]. (**c**) Six-fold photovoltage pattern as a function of *V_TG_* and *V_BG_* [257].

When the light is absorbed in an organic solar device, excitons (closely bounded electron-hole pairs) are created in the active layer. To create a voltage in the device, these excitons have to be separated into free charges in a donor/acceptor interface and then accumulate near electrodes. Liu et al. have reported an organic graphene solar cell device using solution-processed graphene film as the acceptor layer. Figure 41a displays a schematic of the device structure [255].

#### 7.3.3. Photo-Thermoelectric Effect

As mentioned before, most solar cells are photovoltaic devices. Nevertheless, solar power can also be collected and generate electricity using devices based on the photo-thermoelectric effect.

It is proved that the generation of photocurrent in the graphene–metal contacts is a result of the photovoltaic effect. However, recent research on the monolayer–bilayer interface indicate that the photo-thermoelectric effect is vital in photocurrent generation. Gabor et al. reported that hot electronic carriers dominate the intrinsic optoelectronic responses from room temperature down to 10 K.

They designed a graphene solar cell based on the photo-thermoelectric effect for generating photovoltage [257]. A local laser excitation on dual-gated monolayer and bilayer graphene p-n junction devices is employed to examine the optoelectronic responses’ characteristic. Figure 41b demonstrates the device structure in the experiment. As shown in the figure, the device contains a global bottom gate and a local top gate. A six-fold photovoltage pattern as a function of bottom-gate (*V_BG_*) and top-gate voltage (*V_TG_*) was obtained using laser excitation at the p-n interface, see Figure 41c. By altering the gate voltage, four regions of n-p, n-n, p-p and p-n were built in the double-layer graphene. The obtained highest optoelectronic response was reported to be 5 mA/W at low temperatures.

### 7.4. Reconfigurable Multi-Function Logic

We are entering an era that the downscaling of Si-based MOSFET devices may reach the physical limit. Many efforts have been made to search for alternative solutions and materials to silicon-based devices. Graphene-based transistors are considered to be a promising alternative to CMOS. However, as discussed before, its zero bandgap and degraded exciting features after introducing a bandgap is the main disadvantage for graphene to be used in switching applications. For instance, the mobility would decrease in the case of GNR materials. Recently, a new approach was developed using doped graphene films for switching applications [258].

**Figure 42:**
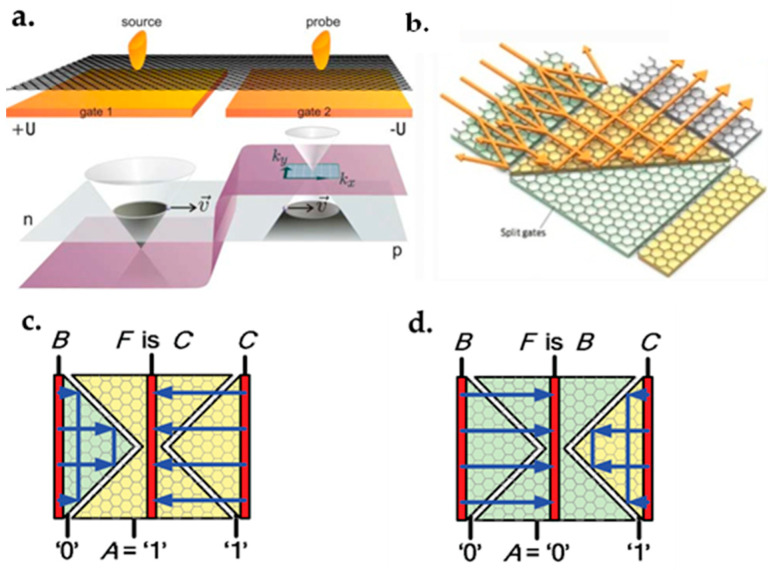
(**a**) The effect of applied voltages (positive and negative) on graphene’s Fermi level [258]. (**b**) Total reflections of electrons when the angle between the incidental electron beam and PN interface is 45° [260]. The schematic of the device when used as an MUX, (**c**) *A* = ’1′, *F* = *C*. (**d**) *A* = ’0′, *F* = *B* [260].

Graphene could be doped by applying an electrostatic voltage [259]. When a positive voltage is applied, the Fermi level would increase and the graphene would behave like an n-type semiconductor. By contrast, the Fermi level drops when applying a negative voltage; thus, p-type graphene would be achieved. A PN junction could be obtained by applying symmetric voltages to two split gates. Figure 42a shows the obtained PN junction with positive voltage on gate1 and negative voltage on gate2 [258].

The electron transmission probability T between the interfaces is largely dependent on the angle θ between the incident electron beam and the PN junction interface. It follows the equation [261]:

(10) $$T\left(\theta \right)=cos^{2}\left(\theta \right)e^{-\pi k_{F}dsin^{2}\left(\theta \right)}$$

where $k_{F}$ is the Fermi wavevector and *d* is the gap.

When θ equals 45° (the critical angle), the electron beams would reflect totally and give an on/off current ratio in the range of 10^3^ and 10^5^ [260]. The total reflection phenomenon is displayed in Figure 42b.

Reconfigurable multifunction logics can be built based on this angular dependence of carrier reflection phenomenon. Tanachutiwat et al. achieved multiplexer (MUX), AND, OR, inverter and buffer functions using this phenomenon [260].

Figure 42c,d display the device consisting of three split-back gates and three electrodes located on the top of the single graphene layer. Symmetric voltages are applied to create PN junctions. In this research, the logic ‘0’ and ‘1’ were defined as $-\frac{1}{2}V_{DD}$ and $+\frac{1}{2}V_{DD}$, respectively. The input *A* was defined by the middle back gate. The other two back gates were connected to ‘0’ and ‘1’ electrostatically doping graphene areas above them to p-type and the n-type, respectively. For the electrodes, the middle *F* was the output terminal. The *B* and *C* were defined as inputs. When *A* = ‘1′, the middle area was n-type. The output electrode *F* would be connected to the right electrode *C*, which means *F = C*. In this case, there would be a total reflection at the *B* and *F* PN junction, where a very high resistance would be created, because of the critical angle as mentioned earlier. Consequently, there is no current flow between the *B* and *F* electrodes.

In the case of *A* = ‘0′, a p-type area would be constructed on top of the middle back gate. Then, *F* was connected to the left electrode. It means *F = B*. Meanwhile, there is no current flow between *C* and *F*. As a result, this device behaves like a MUX function:

(11) $$F=AC+\bar{A}B$$

Figure 42c,d demonstrate the device working as an MUX. The device can also be turned into other logic devices by modifying the input voltages.

This advantage of graphene-based CMOS over traditional Si-based CMOS makes it a promising option to build smaller microelectronic devices and to further continue Moore’s law. Moreover, these devices have better delay-power product and signal restoration compared to traditional CMOS devices with similar device footprints [260].

### 7.5. Graphene Biosensors

**Figure 43:**
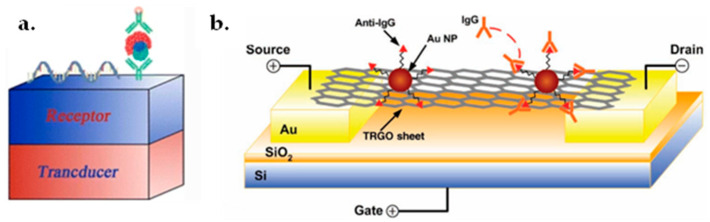
(**a**) Illustration of a biosensor, that is created with a receptor and a transducer. (**b**) The illustration of GFET detecting interactions amongst anti-IgG and IgG biomolecules [268].

A biosensor is a device used to detect the presence of a biological component like molecules. It is based on sensing the interaction with some other known bio-feature and producing a detectable signal. A general biosensor contains a receptor where the molecule interactions happen and a transducer detecting the interactions, as displayed in Figure 43a. The two of the most sensed interactions detected with biosensors are the antibodies/antigens and the single strands of DNA. Carbon-related materials, such as carbon black [262], graphite [263], and CNTs [264,265], have been widely applied in biosensors because of their biocompatibility [266,267]. Nevertheless, the application of graphene as transducers in biosensors has just begun. It is reported that graphene has many advantages compared to other carbon materials such as CNTs. For example, graphene offers a larger sensing area concerning CNTs. Moreover, exfoliated graphene has a higher sensitivity due to the elimination of metallic catalysts such as Ni, which commonly exist in CNT-based biosensors. Different types of graphene biosensors have been reported recently, such as bio-FETs [268,269,270,271,272], electrochemical biosensors [273], impedance biosensors [274] and fluorescence biosensors [275].

#### 7.5.1. Graphene Bio-FETs

A bio-FET is a device detecting interactions between molecules at the FET’s channel or gate [270]. The charge density at the gate region would change when the interactions happen owing to the redistribution of charge carriers in the channel. This will lead to a conductivity shift of the device. This detecting approach is label-free [276], and the procedure is simpler compared to other label approaches, for example, ELISA [277]. Thus, bio-FET has advantages in sensing charged molecules. It seems that the chemically modified graphene is one of the most promising options in the applications for sensing DNA molecules in which the phosphate atom can be charged [271,278]. Nevertheless, the interactions are not stable because of the weak π-type interactions between the pristine graphene and the molecules. Consequently, graphene has to be modified to obtain a tight bond. Oxidation is one of the modification methods. Graphene oxide contains different kinds of functional groups, such as hydroxyl, carboxyl and epoxy. These functional groups can withstand the hydrogen bonding, the covalent or electrostatic attractions [279]. After oxidation, a reduction process was used to obtain graphene layers with such defects, since we are only interested in graphene other than the oxides. The obtained reduced graphene oxide (RGO) FETs have a sensing limit of close to 2 nano molars (nM), comparable with other label-free sensors. The achieved graphene is labeled as thermally reduced graphene oxide (TRGO).

Additionally, other graphene bio-FETs have been reported which are used to detect antibodies or antigens [268,270]. One of the most sensitive TRGO bio-FETs have been fabricated by Mao et al. who exploited Au nanoparticles as their sensing area on TRGO. The sensing limit is in the order of ng/mL when they link an anti-IgG, a type of immunoglobulin antibody in human blood, to TRGO sheets with Au nanoparticles in the sensing experiments. Figure 43b schematically demonstrates the TRGO biosensor.

#### 7.5.2. Impedance Biosensors

An impedance biosensor is used to sense the interaction of biomolecules based on detecting the shift of impedance at the sensing area. Because nanomaterials have high sensitivity to the surrounding electronic variations, sensitive devices can be fabricated by detecting the biomolecules electrically. For instance, specific proteins can be detected with a graphene-based impedance biosensor [274,280,281].

Bonnani et al. and Pumera et al. [280] claimed that pristine graphene is promising in the application for sensing Hairpin-DNA (HpDNA) molecules. The experiment was carried out by fixing Hp-DNA on monolayer and bilayer GNRs with the physically adsorbing method. They have demonstrated that when the complementary single-strand DNA (ssDNA) causes a hybridization, a partial release of HpDNA happens. As HpDNA is negatively charged, the release will cause a decrease in the charge density at the electrode surfaces. As a result, the impedance decreases. They have identified the diminishment of the impedance because of the hybridization with an accuracy of 82 pM [280].

**Figure 44:**
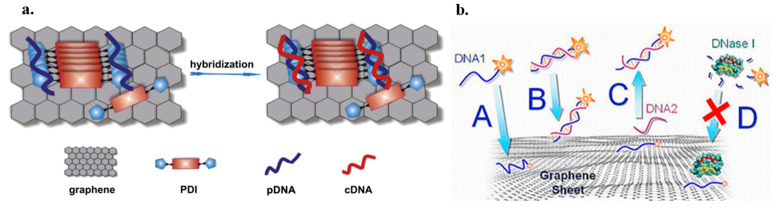
(**a**) Illustration of DNA hybridization on the PDI/graphene platform [274]. (**b**) A schematic of fluorescent-tagged DNA hybridization: A stands for no hybridization occurring when an irrelevant ssDNA interacts. B stands for no absorption of double-stranded DNA on graphene. C stands for a complementary ssDNA hybridization and fluorescent emission [275].

Recently, Hu et al. have successfully fixed a positively charged N, N-bis-(1-amino propyl-3-propylimidazolium salt)-3, 4, 9, 10-perylene tetracarboxylic acid diimide (PDI) to the graphene films. The ssDNA is anchored due to the electrostatic interaction between the positively charged PDIs and the negatively charged DNA molecules. They employed a similar method, in which no partial release happens and no attraction is disturbed, to detect the hybridization, as shown in Figure 44a [274].

Gas sensors can also be fabricated using graphene [282] and the detecting procedure is similar to that of impedimetric biosensors. In other words, the adsorption of particular gas molecules would cause a change in impedance [283].

#### 7.5.3. Electrochemiluminescence Biosensors

Electrochemiluminescence biosensors can identify molecules based on detecting the emission light from an electrochemical reaction. Tang et al. reported the immobilization of fluorescent-tagged ssDNA on functionalized graphene. They successfully detected the fluorescent light after introducing the complementary ssDNA into the solvent, confirming the hybridization of DNA strands, as shown in Figure 44b. Moreover, they found that the ssDNA is stable on graphene because the deoxyribonuclease I fails to degrade the adsorbed ssDNAs on graphene [275].

### 7.6. Graphene Optoelectronics Applications

#### 7.6.1. Graphene Photodetector

Graphene also has many advantages in the fabrication of optoelectronic devices, such as the production of multiple electron-hole pairs with a high-energy excitation and high mobility [95]. However, the main disadvantage is the low optical absorption, resulting in a low responsivity.

When a light laterally incidents on the graphene film on a waveguide, the light absorption is determined by the film length (or the waveguide length). A full absorption happens providing a long enough film length.

**Figure 45:**
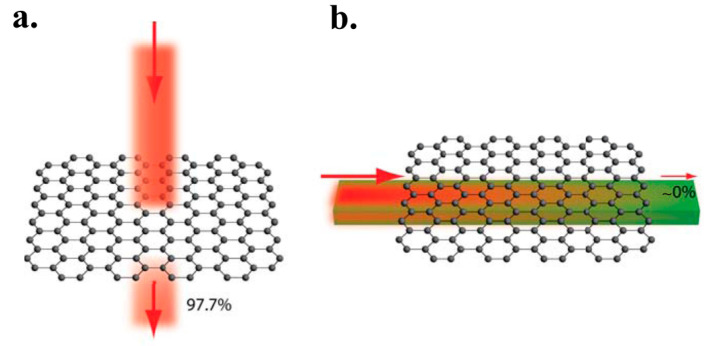
(**a**) The absorption coefficient is 2.3% when the light vertically incidents on a graphene sheet. (**b**) The light laterally incidents on a graphene sheet where the interactions with light are determined by the graphene sheet length [284].

Graphene absorbs the incident light in both vertical and lateral directions, as demonstrated in Figure 45. The oxide layers are employed for the isolation when transferring the graphene sheet on a Si-based waveguide [284].

**Figure 46:**
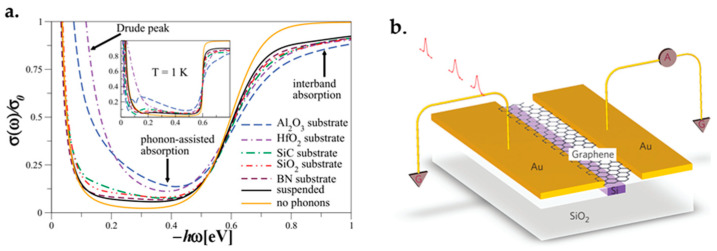
(**a**) Calculated frequency as a function of the optical conductivity on different substrates using a 5 × 10^11^ cm^−3^ impurities concentration and 0.3 eV chemical potential [285]. (**b**) The structure of the graphene-based photodetector [286].

The vertical light is absorbed via the inter-band and intra-band transitions, see Figure 46a. The conductivity curve could deviate from the universal value of 2.3%. The curve is dependent on the substrate material, because of the phonons which change the inter-band absorption behavior [285].

To increase the device’s photo responsivity, it is mandatory to search for new isolation material [286,287]. It should be noted that other 2D crystals, such as WS_2_, black P and MoS_2_, are expected to gain high responsivity for future photodetectors [288,289].

Gan et al. have proposed the integration of graphene photodetectors on silicon-on-insulator (SOI) bus waveguides to overcome the drawbacks of the low photo-responsivity. In their study, they successfully obtained a more than 0.1 AW^−1^ photo-responsivity with a uniform response between 1450 and 1590 nm by increasing the graphene’s absorption. The device structure consists of one waveguide and two Au electrodes sitting at the opposite sides of the waveguide and collecting photocurrents, see Figure 46b. Moreover, the graphene layer is isolated from the beneath Si using a 10 nm SiO_2_ layer. The graphene utilized here is a 53 μm double-layer graphene whose light absorption is two times larger than that of single-layer graphene. It is metal doped at the junctions. It should be noted that the coupling between the graphene and the waveguide is evanescent [286].

**Figure 47:**
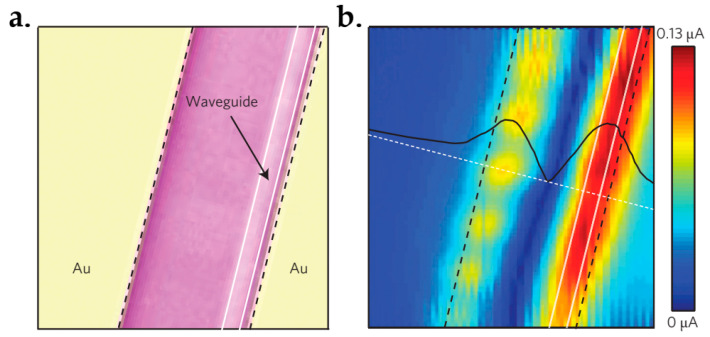
(**a**) SEM image of the measured region. (**b**) Photocurrent diagram at zero bias voltage. Two photocurrent strips show at the metal/graphene interface [286].

They measured the photocurrent by illuminating the sample from the top using a continuous wave laser at the length of 1550 nm at zero bias voltage. Figure 47a displays the device’s reflective scanning image, and Figure 47b shows the measured photocurrent pattern at zero bias voltage. The highest photocurrent obtained is about 13 μA at the waveguide region, corresponding to 50 μW excitation power. Because of the graphene photodetector’s low detecting efficiency at normal illumination, a poor responsivity of 2.6 × 10^−4^ A W^−1^ was obtained.

**Figure 48:**
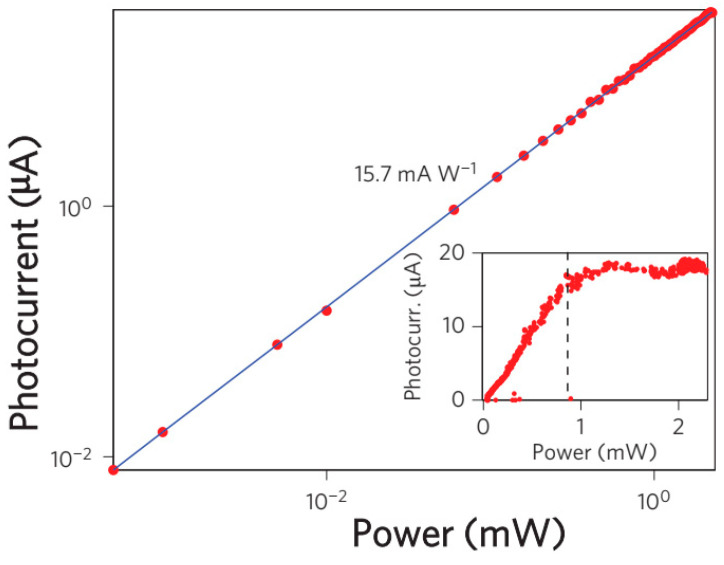
The photocurrent as a function of the continuous wave laser power at zero bias. Inset: photocurrent as a function of the pulsed OPO laser power at 2000 nm wavelength [286].

The photodetection efficiency was measured by modulating a 1550 nm continuous-wave laser at a low frequency through a pre-amplifier and a lock-in amplifier. Figure 48 displays the photocurrent (*I_photo_*) as a function of the input power (*P_input_*) at zero bias. The calculated responsivity (*I_photo_/P_input_*) was 15.7 mA·W^−1^, which is two orders of magnitude larger compared to that of the normal incidence. This is owing to the increased interactions between graphene and light. Moreover, efficient separation of photoexcited electron-hole pairs because of the strong local electric fields along the metal-doped junctions also plays a role.

#### 7.6.2. CMOS-Compatible Graphene Photodetector

Optical interconnection and communication as alternatives to electrical wiring are playing an important role in linking intra-chip and inter-chip communication [290]. Especially, it is more attractive to integrate with CMOS technology because it will be cost-effective to integrate optics and electronics on one chip. To do this, both the integration of Si and the optical absorption materials are needed, where the Si is used to realize the optical waveguides and the graphene is used as the absorption material due to its optical characteristics [28,291,292,293,294].

Graphene was firstly found to be able to use as a photodetector when utilized in a back-gate transistor [295,296,297]. A generated current was discovered at the metal–graphene interface due to the band bending. Additionally, photocurrents were also observed at the interfaces of monolayer and bilayer graphene and p-n junctions due to the thermoelectric effect [298,299].

**Figure 49:**
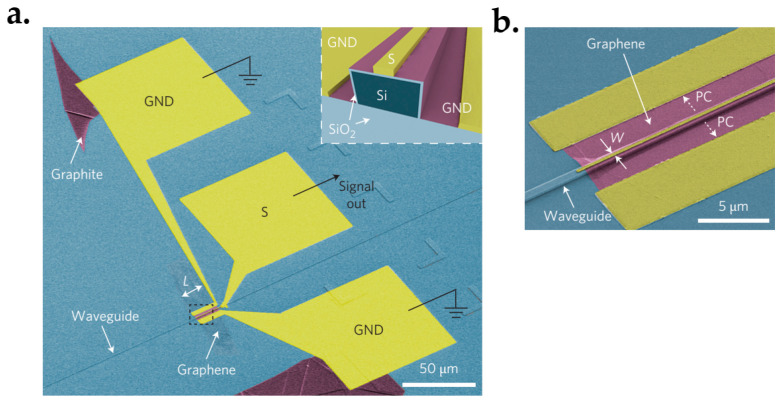
(**a**) Graphene photodetector integrated on the waveguide. The purple part is the graphene’s active region. Inset: graphene covers the sidewalls and the topside. (**b**) The magnified region of the black dashed area in (**a**). The photocurrent is from central electrode *S* to the ground (GND) [300].

Pospischil et al. have reported the design and fabrication of a CMOS-compatible graphene photodetector device [300], as shown in Figure 49. The fabrication process has three steps. It starts with the Si waveguide’s etching and passivation. This follows with the graphene deposition. The last is metallization.

When the device operates, the photocurrent is generated toward GND because of the potential gradient existing between the electrode S and the graphene. The potential gradient is due to the different doping levels in the metal-uncovered and covered graphene parts. Because of the zero bandgap in graphene, it has a high bandwidth photodetection.

**Figure 50:**
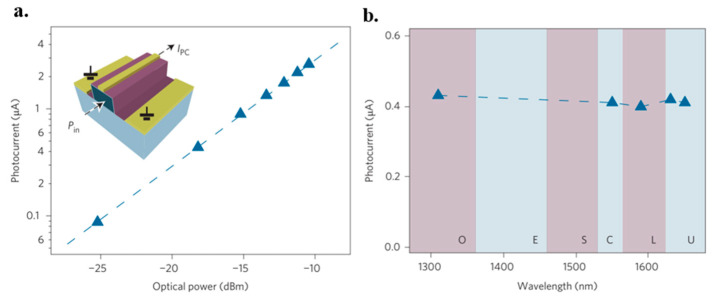
(**a**,**b**) show the photocurrent as a function of the optical power and the wavelength, respectively. The triangles plots are the measured data [300].

The photocurrent was measured through a transimpedance amplifier with low impedance connecting to the device. Figure 50a displays that the photocurrent is linearly dependent on the optical power. Figure 50b shows the bilayer graphene photodetector’s photocurrent as a function of the wavelength. A flat response is observed from 1310 nm (O band) to 1650 nm (U band). The ultra-wide range of wavelengths in which the inter-band optical transitions take place is owing to the graphene’s gapless feature [301,302,303,304].

Except for the wideband operation and the compatibility with CMOS, graphene also has other features, such as low-energy consumption, high-speed operation, cost efficiency, small footprint and simplicity, which can be used for optical interconnects.

#### 7.6.3. Graphene-on-Graphene Modulator

**Figure 51:**
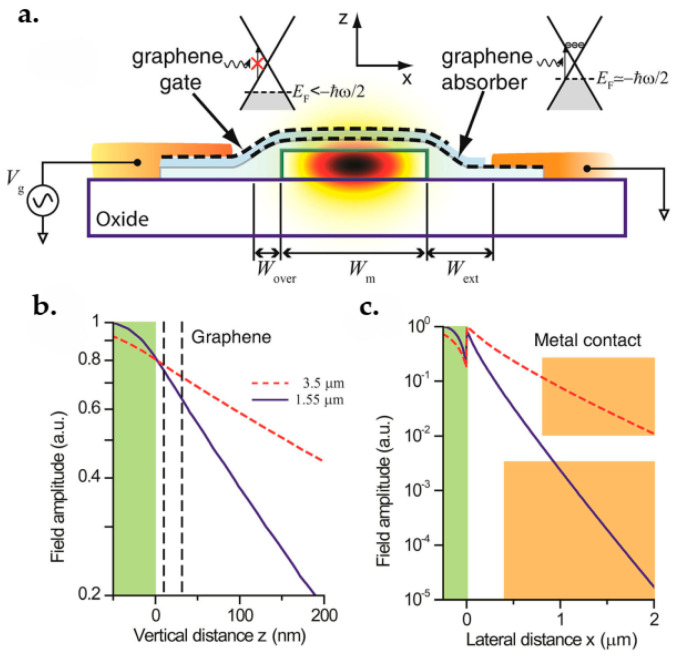
(**a**) Waveguide-integrated bilayer graphene modulator. The overlay displays a typical waveguide’s fundamental TE mode profile [305]. (**b**) The electric field amplitude decreases from the waveguide top. (**c**) The field amplitude decreases from the waveguide side [305]. In (**b**,**c**), the green part is the waveguide, the black dashed lines represent the graphene layers and the orange parts are the metal contacts.

The linear absorption coefficient and the absorption wavelength of graphene can be tuned via a gate voltage. This research was undertaken by Koester et al. using a graphene-on-graphene modulator [213]. The modulator was fabricated using the CVD-grown graphene on a planar single-mode photonic waveguide. Figure 51a shows the device structure. The downside graphene layer acted as the absorber. It was separated from the beneath waveguide by an oxide layer grown with the atomic layer deposition method. The Ohmic contacts were fabricated with the graphene patterning and the metal deposition. This followed with a deposition of another oxide layer on the graphene. Then, the steps were repeated to grow the second layer graphene and the contacts. The obtained device is simple and has a high modulator speed and low insertion loss. Moreover, it can modulate in the wavelength range from near-infrared to mid-infrared.

The electrical field amplitude as a function of the waveguide length in both the vertical and the lateral directions are displayed in Figure 51b and Figure 51c, respectively. The modulator can work appropriately by adding a DC offset voltage at a specified background carrier concentration range. In this situation, the top graphene layer is transparent at the working wavelength and the bottom graphene layer acts as an absorber.

Simulations have been done to investigate the modulator’s performance by utilizing an equivalent circuit model, which is based on random potential fluctuations, quantum capacitance effects and Fermi-Dirac statistics. The carrier concentrations in the graphene are calculated through the integration of the Fermi-Dirac distribution function with the graphene density of states, where a random potential with a standard deviation is applied to the graphene band structure. The mean electron and hole sheet densities and the mean absorption probability are achieved by averaging over 1000 random values.

**Figure 52:**
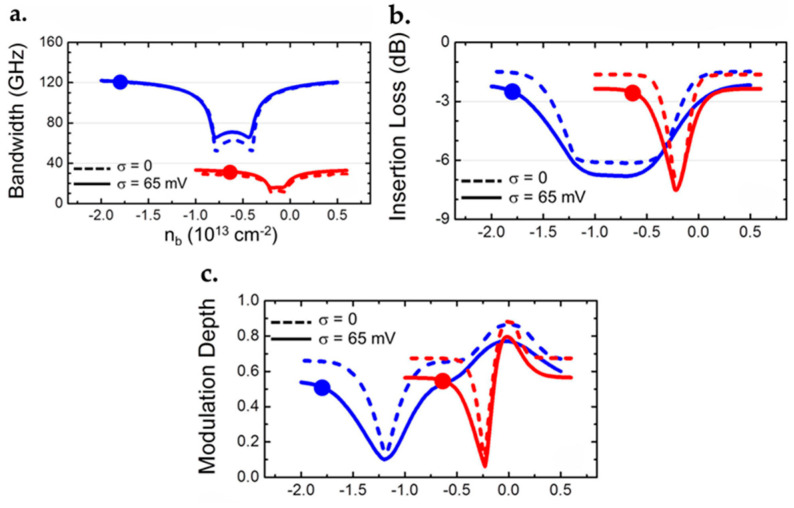
(**a**) The bandwidth, (**b**) the insertion loss and (**c**) the modulation depth as a function of the background electron concentration. The blue and the red are 1.55 and 3.5 µm for graphene, respectively. The dashed lines and the solid lines are with two standard deviations of *σ* = 0 and 65 mV, respectively. The circles are the optimal design points [305].

The results are displayed in Figure 52. The simulated parameters such as the bandwidth, the insertion loss and the modulation depth are calculated as a function of background carrier concentration with two standard deviations of *σ* = 0 and 65 mV, respectively.

To obtain the bandwidth using Equation (12), the total capacitance and series resistance are required. The total capacitance is a series combination of the oxide capacitance and the quantum capacitances of the two graphene layers. The former is calculated using a simple parallel plate approximation. The latter can be calculated from the derivative of the charge concentration with respect to the Fermi-level position. While the total series resistance can be calculated by adding together the resistances of the two graphene layers and the metal contacts. The calculated bandwidths are displayed in Figure 52a.

(12) $$f_{3\mathrm{dB}}\;\left(\mathrm{Bandwidth}\right)=\frac{1}{2\pi \tau_{RC}}$$

To obtain the insertion loss and the modulation depth, the total absorption coefficient and transmission (*T*) shall be calculated firstly. The total absorption coefficient was calculated using the sum of the entire structure (*α*) and the additional attenuation coefficient (*α*_m_). The *α* is the sum of the absorption coefficients in the two graphene layers, while the *α*_m_ is the absorption coefficient due to the metal contacts. The total transmission (*T*) can be calculated with the total absorption coefficient according to Equation (13). The *T_max_* and *T_min_* are the maximum and minimum of the *T* which were obtained from one full AC voltage cycle. With these two values, the insertion loss and the modulation depth can be calculated according to Equation (14) and Equation (15), respectively [305].

(13) $$T\;\left(\mathrm{Total}\;\mathrm{Transmission}\right)=e^{-\left(\alpha +\alpha_{m}\right)L_{m}}$$

(14) $$L\;\left(\mathrm{Insertion}\;\mathrm{Loss}\right)=10\log\left(T_{max}\right)$$

(15) $$M\left(\mathrm{Modulation}\;\mathrm{Depth}\right)=\frac{T_{max}-T_{min}}{T_{max}}$$

### 7.7. Graphene Photo Memtransistor

**Figure 53:**
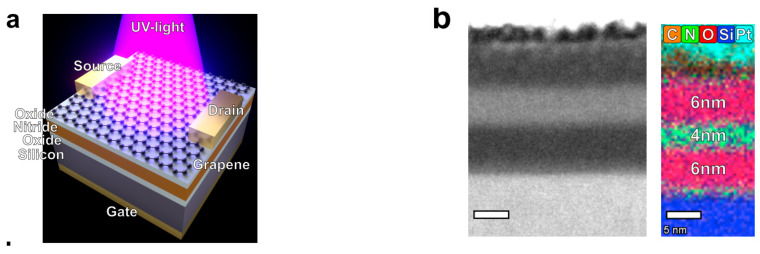
(**a**) Schematic of the graphene memory device. (**b**) STEM dark field image and X-ray elemental composition image of the device and the elemental composition. The scale bars are 5 nm [307].

**Figure 54:**
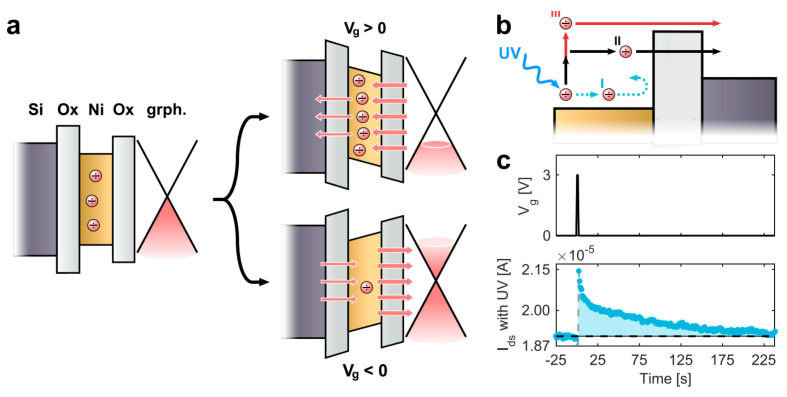
(**a**) Schematic of the tunneling and charge trapping process (**b**) Schematic of how UV light detraps electrons. (**c**) Carriers’ trapping and detraping process [306].

A very recently developed application is the graphene photo memtransistor. Figure 50 illustrates the structure of the graphene memory device. Note that the top platinum layer is not related to the real device geometry but for the assistance to cut out the sample for STEM imaging during the FIB process. The graphene can be integrated with conventional flash memory technology. This integration can achieve programmable doping of graphene by applying short gate pulses, setting the graphene’s conductivity to either a higher or a lower conductive state. As shown in Figure 53, a positively applied bias on the gate electrode makes the electrons tunnel from the graphene into the nitride layer; thus, p-doping graphene. Likewise, a negative bias will result in n-doping of the graphene, as shown in Figure 53a. The carriers are able to de-trap by using ultraviolet light, as illustrated in Figure 54b. Figure 54c shows that the conductivity initially goes up instantaneously after applying the pulse, but then drops back off gradually to the initial level. The electrons gain their energy from the UV light source and are being expelled from the nitride layer due to the internal field generated by the other trapped charges. The approach may be used for integrating graphene in CMOS technology memory applications, or even could be suitable for large-scale neuromorphic computing structures [306].

### 7.8. Other Applications

It should be noted that the above-mentioned are not all applications of graphene. There are numerous applications of graphene which have been reported in many articles.

The graphene supercapacitor is another example of a graphene application. Promising results have been reported when it was used as batteries [166,307,308,309,310,311]. Moreover, adhesive conductive film recently has been achieved using graphene films decorated with silver nanoparticles [312]. The smallest 2D resonators with megahertz range frequencies were recently fabricated on the suspended graphene [313]. Other applications such as photocatalytic behavior have also been investigated in some studies [314]. The last but not the least example is the application in infrared photodetectors. A strong photo-response near metallic electrodes was reported [29,315,316].

## 8. Applications of CNTs

CNTs have brought much interest because of their amazing mechanical, electrical and thermal features [317,318,319]. These extraordinary properties guide scientists to utilize CNTs in a wider scope of operations, for instance, micro-electronics and nano-electronics, spintronics, optoelectronics, material sciences, energy storage and mechanics. This section provides a review of these applications especially based on recent works related to the chemical and electrical properties of CNTs.

### 8.1. Structural Applications

CNTs with remarkable properties and qualities related to their structure could find growing structural applications [320,321,322,323]. Textiles and fibers based on CNTs can produce waterproof or bullet-protecting jackets [324,325,326,327]. Moreover, CNT composites with polymers such as polyethylene provide more elasticity than polyethylene alone. Another structural application of CNTs is utilizing them to replace metals to make lighter and stronger constructions for bridges, flywheels and fire protection elements [53].

### 8.2. Electromagnetic and Electronic Applications

Buckypaper (buckytubes) of CNTs has a strong and light-sheet structure and can be used as a heat sink of chipboards and backlight of LCD screens and protect electrical devices (Faradaic cage) [328,329,330,331]. Another exciting use of CNTs is the ability to generate powerful magnetic fields. For this purpose, MWCNTs are coated with magnetite and utilized in many applications [38].

This attractive material demonstrates unique electronic properties, so it has been one of the great elements for building electronic devices such as diodes, transistors and interconnectors.

### 8.3. Transistors

**Figure 55:**
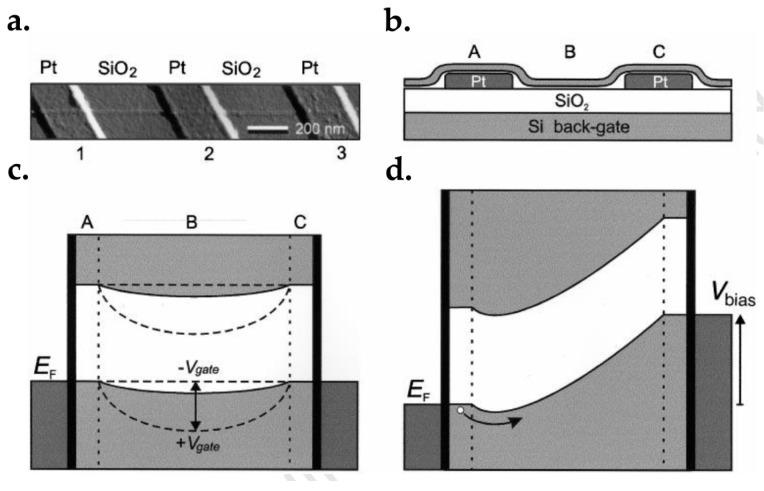
(**a**) AFM image of a single SWCNT over Pt electrodes. (**b**) Schematic view of the back-gate CNTFET reported by Tans et al. Band diagram of the proposed FET (**c**) without bias voltage (i.e., $V_{DS}=0$, off state) and (**d**) with bias voltage (on sate) [332].

**Figure 56:**
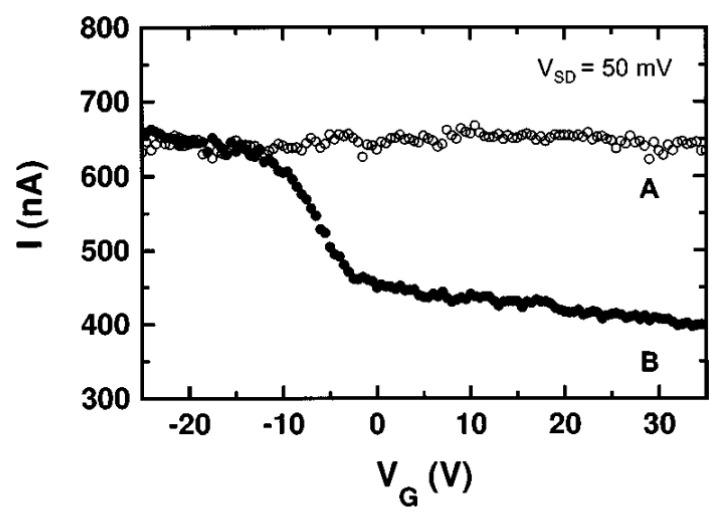
Curve A belongs to a typical MWCNT-FET, as no apparent switching is observed. Curve B belongs to a deformed MWCNT-FET. This device shows a faint switching action, and there is a significant current in the off state due to the metallic behavior of the MWCNT channel [333].

Amongst the many full of potential applications of CNTs is the realization of carbon nanotube field-effect transistors or CNTFETs as a replacement for silicon-based FETs. The first CNTFET was reported in 1998, with a conventional MOSFET-like back-gate structure shown in Figure 55a,b, indicating that CNTFETs could be potential successors to silicon FETs [332,333]. However, such CNTFETs tend to exhibit p-channel behavior. This is not an intrinsic characteristic of CNTs but rather attributed to source/drain metal contacts [54,334,335]. Theoretically speaking, the CNT bandgap is inversely proportional to its diameter. Therefore, MWCNTs which have bigger diameters than SWCNTs are not the prime candidates for an effective semiconducting channel. In other words, CNTFETs utilizing MWCNTs as their channel do not demonstrate transistor functionality. Martel et al. [333] reported that deformed MWCNTs show a faint switching action (see Figure 56) and may be used as an FET channel since MWCNTs are easier to fabricate.

Since every transistor implemented would have the same gate voltage, a back-gate FET has the disadvantage that it does not allow the fabrication of multiple transistors on a single chip. A novel implementation technique was proposed by Bachtold et al. [336] to eliminate this constraint.

**Figure 57:**
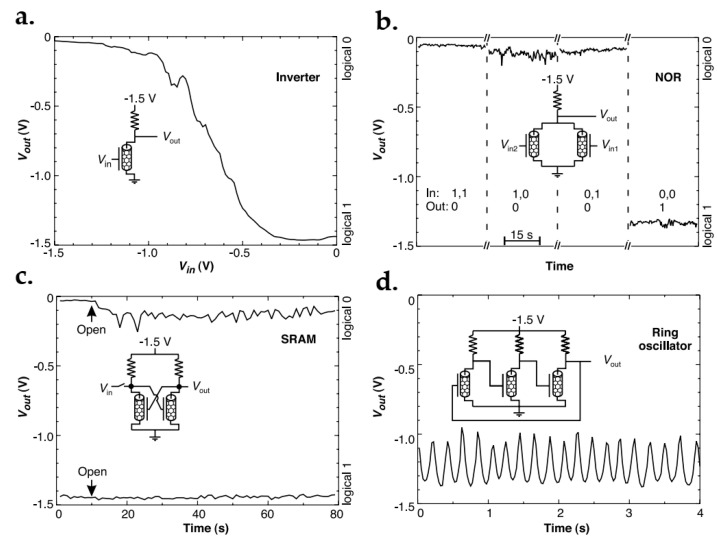
(**a**) VTC of the RT (resistor transistor) NOT gate. The test results of the (**b**) NOR gate, (**c**) bi-stable storage element and (**d**) ring oscillator [336].

**Figure 58:**
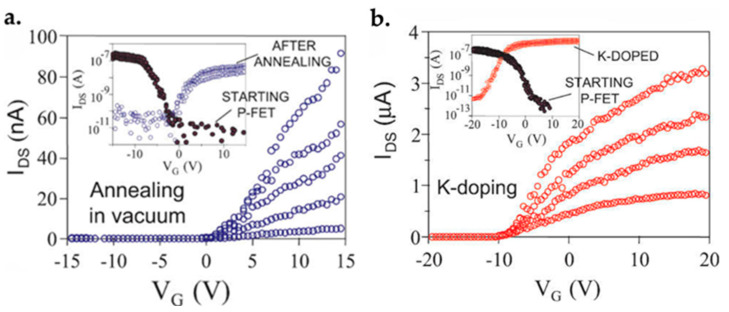
(**a**) Vacuum annealed p-FET exhibits n-type behavior. (**b**) Potassium doping of p-FETs, note that there is a change in location in the threshold voltage after doping [337].

To integrate multiple transistors on a single chip, a resistor-transistor logic was introduced. In this logic style, the negative voltage is regarded as the logic value 1 while the zero voltage is considered to be the logic value 0. Figure 57 illustrates the measured results of NOT, NOR, front-to-back coupled NOT gates and a ring oscillator circuit. However, a complementary logic gate set is more desirable, which requires n-type CNTFETs. As stated before, such CNTFETs exhibit p-type behavior. This problem can be resolved by doping the SWCNTs with an electro-positive dopant (e.g., potassium) [334,337] or by vacuum annealing [54]. The acquired n-type CNTFET can revert to p-type if exposed to oxygen [60]. The I-V plot diagrams of back-gate CNTFETs are depicted in Figure 58.

**Figure 59:**
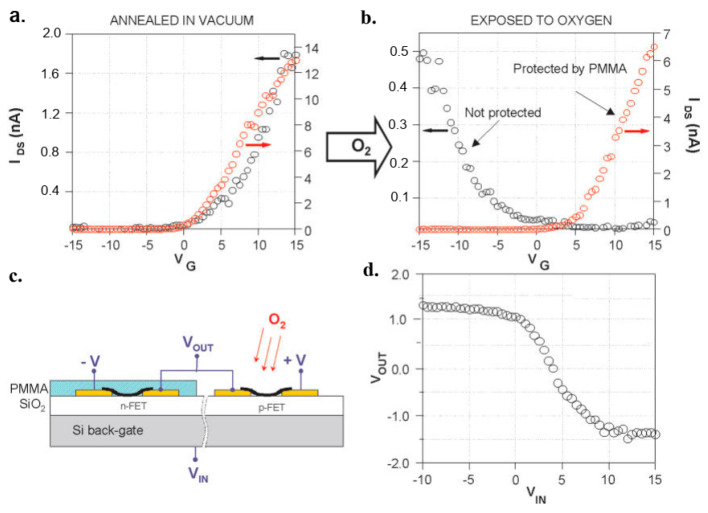
(**a**) Two vacuum-annealed CNTFETs, the red curve belongs to an FET that PMMA protects. (**b**) Unprotected (gray curve) CNTFETs revert to p-type after the oxygen exposure. (**c**) The n-CNTFET and p-CNTFET are wired together to form an NOT gate. (**d**) VTC curve of the intramolecular NOT gate [337].

**Figure 60:**
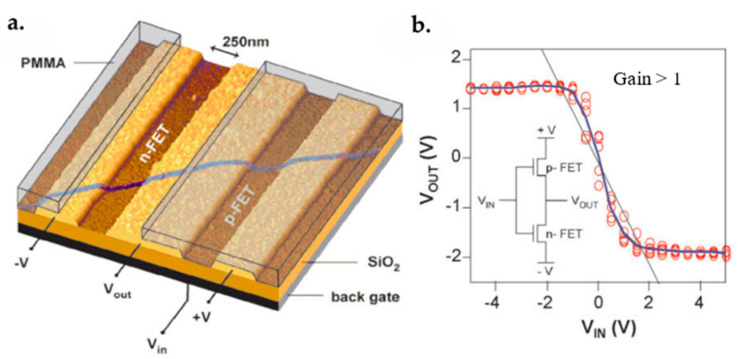
(**a**) Illustration of the intramolecular logic gate, two p-type CNTFETs are introduced in series using the single nanotube bundle which is placed on the gold electrodes. (**b**) Characteristics of the inverter, red dots are the raw data and the blue line indicates an average of all measurements (V =± v) [337].

Furthermore, PMMA is used to insulate the n-type CNTFET from oxygen/air exposure (see Figure 59) [337]. However, an intramolecular NOT gate fabrication is explained in the sequence depicted in Figure 59c. Intermolecular fabrication (i.e., using the same nanotube for both transistors utilized in a NOT gate) is more desirable. The illustration and the result are shown in Figure 60. Another example of such logic element capable of integration was published by Liu et al. [338].

CNTFETs discussed so far are categorized as Schottky barrier (SB) FETs since the switch was controlled mainly by contact resistance rather than CNTs’ conduction. The key to improving the performance and the conductance modulation lies in providing better contacts (usually high work function metals such as gold are used) [339,340]. The back-gate structure reported here has two significant issues. First, the integration of many transistors on a single chip is troublesome, which was addressed by Bachtold et al. [336]. Second, the gate-to-substrate dielectric constant is reduced due to the practiced open-air structure. Therefore, a top-gate structure is favorable [9]. The method presented by Wind et al. [339] also enables in situ modification of p-type FETs to produce n-type FETs.

**Figure 61:**
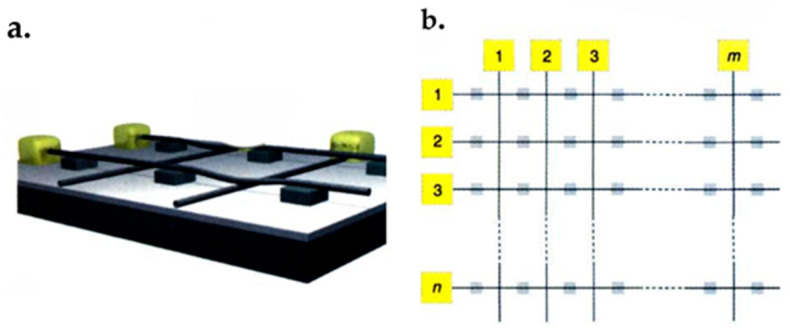
Suspended CNT device architecture. (**a**) A 3D schematic of the suspended crossbar array in which four of the contacted junctions are in ON state and the other separated junctions are in OFF state. (**b**) Top-view of the n.m array devices, yellow blocks are metal electrodes and grey blocks are suspended nanotubes [341].

A design for a non-volatile RAM (Random Access Memory) was proposed by Rueckes et al. [341], using a matrix of SWCNTs. One set of SWCNTs is placed on a substrate while another perpendicular set is placed on top of them with the assistance of some evenly placed supports (see Figure 61a,b). However, rather than a conventional bi-stable design, each cross-over of nanowires in this design represents a bit. The off-state is defined by the high electrical resistance (i.e., the free suspension of nanowire), while the on-state is defined by the low resistance of contact junction when drawn together (nanowire cross-over). It is achieved by charging the two corresponding nanowires with different signs [11]. Another memory device with high mobility was reported by Brintlinger. The applied gate voltage of the device was swiping in the range between +10 and −10 V [342]. This is interesting because it suggests the injection of charge into the dielectric.

**Figure 62:**
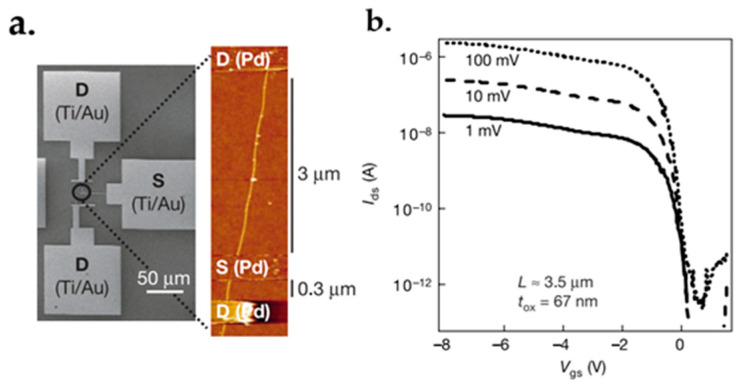
(**a**) SWCNT devices with Pd-contacts on SiO_2_/Si. (**b**) Transfer characteristics of the device with pure Pd-contacts [343].

As mentioned above, the CNTFET’s performance is reduced by the SB in the vicinity of contact electrodes. Therefore, extending the potent ballistic or near ballistic transport characteristic of SWCNTs will provide better contacts and wetting interaction with the SWCNTs utilized in FETs. SWCNTs are expected to show two units of quantum conductance which gives an electrical resistance of 6.5 kΩ. This makes the SWCNTs a potential material for the realization of ballistic FETs. Pd was suggested as a suitable candidate for contact electrodes (see Figure 62a). Forming SB at the contacts is dependent on the SWCNT’s bandgap and the contact’s work function. The modification of the Pd work function revealed that reduced Pd work function increases the SB height and limits the device’s performance (Figure 62b). Additionally, SWCNTs with small diameters (~2 nm) form immensely low SBs, though they are not entirely removed [343].

### 8.4. Diode

Besides transistor construction, photodetectors are a valuable field to employ semiconducting CNTs in nanoelectronics. As a fundamental building block of semiconducting electronics, the diode is one of the structures that can be formed based on CNTs instead of Si p-n junctions [344,345]. The quasi one-dimensional nature of the CNT leads to the dominating photocurrent spectra by photo-excitation, along with hundreds of mV of energy [346,347,348]. The high excitation binding energy of the CNT-based photodetectors makes the phonon-assisted excitation dissociation negligible [349,350].

A diode made of CNTs was reported by Bughes et al., who described a diode with Pd and Ti contact split gates [351]. This device displayed strong rectification and ultra-low leakage current. Additionally, many studies have been performed using CNT-based diodes in the power supply applications such as mobile communication devices. These devices invert the diode’s reflection direction in the high-speed signal process [352,353].

### 8.5. Interconnection

In order to manage the heat of IC, it is necessary to replace conventional conductors with high electrical resistance and the Joule effect [354]. Meanwhile, increasing temperature limits the electrical current in conventional interconnections. CNTs with outstanding properties are a great alternative conducting material for this purpose [355,356]. Several reports are based on employing CNTs for interconnection production. The results demonstrated the possibility of obtaining a negative temperature coefficient of the resistance [357,358,359,360].

### 8.6. Sensor and Biosensors

Recent investigations are focusing on dealing with the design and fabrication of sensors and biosensors using CNTs. Most of its chemical and electrochemical properties are related to this unique large surface area [361]. The absorption of molecules on the CNTs surface changes the electronic properties and makes them a promising starting material for developing sensitive sensors and biosensors [142,143,144,145]. The principle of sensor operation may change the I-V curve of the CNTs due to accumulation or binding molecules on their surface.

Biosensors are analytical devices that provide an analytical response related to a biological interaction using a suitable transducer [362]. An ideal biosensor needs to have excellent stability, sensitivity and wide dynamic range. Therefore, CNTs are a promising material to fabricate biosensors due to their physicochemical characteristics, such as good electron transfer, mechanical strength, extended surface area, biocompatibility and tailorable surface to be functionalized with biomolecules [188,362].

CNT-FET-based biosensors are usually designed based on the CNTs’ conductivity as the transistor channel that connects the source and the drain. Biological interaction near chemical conductive can change the conductivity [147,148,149,363]. Various functionalization methods have been developed to improve the CNT’s dispensability and give access to chemical reagents consequently. These methods make CNTs able to integrate with biological elements and gain electroanalytical properties [364,365].

Covalent binding may result from amine or acid bonding of amino and carboxyl groups [366,367,368,369]. The noncovalent immobilization also can be applied to fabricate biosensors derived from the CNT *π-π* conjugation. The *π* which stacks between the CNT and baroreceptor leads to combining them and fixing biomolecules on the surface of the CNT [370,371,372,373].

One of the most widespread types of CNT-based sensors and biosensors are electrochemical devices. These sensors are based on the CNT modified working electrodes [48]. A redox polymer usually acts as the catalyst that transfers the electron between the base of the electrode and the biomolecules. This arrangement of the CNT and the catalyst improves the electrical conductivity of the CNTs. The catalyst polymers provide reversible redox reactions and thus detect different analytes such as nitride, sorbitol, glucose, hydrogen peroxide, uric acid, dopamine, etc. [374,375,376,377].

### 8.7. Other Applications

Research has been undertaken to develop CNT/metal oxide composites with the ability of electrochemical energy storage. A supercapacitor or electrochemical double-layer capacitor provides a higher energy density than conventional capacitors. These capacitors also have higher power density than batteries and faster charge and discharge rates, representing distinctive superior properties [378,379,380]. Despite these outstanding characteristics, low specific capacitance needs to be modified using combining metal oxides such as MnO_2_, Co_2_O_4_, RuO_2_, NiCo_2_O_4_, etc. [381,382,383,384].

Furthermore, the excellent chemical and physical properties of CNTs make them a proper possibility to be utilized in solar cell structures. CNTs could be an interesting, exciting alternative hole-transporting layer and counter electrode material in different solar cells [385,386,387,388,389]. In addition, they can represent ballistic electrical conductivity, semitransparency and flexibility in solar cell fabrication. CNTs are incredibly fascinating in transparent electronics [390], smart drug delivery [391] and high-power converters [392].

## 9. Conclusions and Outlook

This article reviewed the structural features and properties of graphene and CNTs, the synthesis and transfer methods, the characterization methods and a few important applications of graphene and carbon nanotubes in electronics, optoelectronics and sensors. The conclusions are summarized below.

Carbon nanomaterials such as graphene and CNTs have various special features due to their unique structures. The discovery of the graphene and CNTs has revolutionized the nanoelectronics industry.

CNT has a 1D structure with a high surface-to-volume ratio feature. As a nanotube is a surface structure, its whole weight is concentrated in the surface of its layers. Because of the special chemical, mechanical, thermal, optical, electronic and electrical characteristics, many efforts are engaged in continuing to improve the synthesis techniques and develop the novel applications, such as energy conversion electrode structures, sensor and biosensor design, gas discharge tubes for telecommunication, screening of electromagnetic waves, batteries, hydrogen storage and composite materials.

Graphene consists of a 2D monolayer structure. Graphene can be used in the electronic and optoelectronic applications by tuning its zero-band gap by applying an external electric field or chemical doping. It also has various novel electrical properties such as the Klein tunneling, quantum Hall effect because the electron movements are confined in two dimensions. The atoms are on the surface, making the properties and electronic band structure of graphene sensitive to environmental interactions, surface curvatures and size. Moreover, the charge carrier concentration has a relation with the magnitude and sign of applying a gate voltage. Graphene produces holes at a negative voltage, while it has electrons with a positive voltage. All these special features make it an excellent candidate in various applications such as electronics, optoelectronics, biosensors, and so on. Thus, it is known as an alternative for silicon technology.

Although the growth techniques have rapidly developed but significant market penetration has not yet occurred in any field. These carbon nanomaterials will continue in a research and prototyping phase for the next few years. A major threat and critical bottleneck for the commercialization is the availability of a suitable supply concerning both quality and quantity at a competitive price [393]. Beyond the cost reduction and capacity expansion of the industry, three interrelated challenges still exist: (a) the perception of their immaturity among potential customers, (b) a lack of reliability and standardization and (c) regulatory hurdles (such as REACH) and associated toxicology concerns. The present status of the global supply industry is characterized by the following major trends: steady demand growth in a low rate, stagnation of global production capacity, slow decay of production cost, the sales strongly depending on the quality and consistency of products.

More focus must be paid on making purified materials with fewer defects and on upgrading the lab-scale processes into technologies applicable in industry. Implementing the various technologies such as silicon technologies with CNTs and graphene will make the process more efficient, increase production, and lessen defects in the application of the electronic field. It is expected that, in the near future, novel techniques will emerge and make the CNTs and graphene more affordable and viable in various fields of applications.

## Figures and Tables

**Table 1:** Bandgap calculations for zigzag nanotubes with different methods.

(*n*_1_, *n*_2_)	(6,0)	(7,0)	(8,0)	(9,0)	(10,0)	(11,0)	(12,0)	(13,0)	(14,0)	(15,0)
Reference [70] (*E_g_* eV)		0.243	0.643	0.093	0.764	0.939	0.078	0.625	0.736	0.028
Reference [70] (*E_g_* eV)	0.21	1.0	1.22	0.045	0.86	0.89	0.008	0.679	0.7	0.0
Reference [73] (*E_g_* eV)		0.79	1.12		0.65	0.80				
Reference [73] (*E_g_* eV)		1.11	1.33		0.87	0.96				
Reference [74] (TB) (*E_g_* eV)	0.05	1.04	1.19	0.07						
Reference [74] (LDA) (*E_g_* eV)	Metal(−0.83)	0.09	0.62	0.17						
Reference [75] B3LYP (*E_g_* eV)	0.00			0.079			0.041			0.036
Reference [75] experimental (*E_g_* eV)				0.80			0.042			0.029
Reference [75] LDA (*E_g_* eV)				0.024			0.002			0.00

**Table 2:** Summary of current methods used to produce graphene.

Method	Sources	Advantages	Disadvantages	References
Exfoliation	Graphite	Simple and high yielding	Not fully purified	[120,121,122,123]
Scotch-tape and drawing method	Graphite	Simple and high quality	Cannot be scaled further, limit in size	[119,124,125]
Sanitation	Graphite powder and flakes	High quality	Low production, time consumption	[126,127,128,129,130]
Reduction	Different carbon sourcesOther activated carbons	Large-area monolayer graphene films onto a variety of substrates	Limited in yield.	[131,132,133,134,135,136,137]
Epitaxy	Metal–carbon solutions or 6*H-SiC*	High quality, mono-/bi-/trilayer graphene	Requires expensive equipment, throughput issue and scaling requires significant effort	[144,145,146,147,148]
Chemical vapor deposition (CVD)	CH_4_ and C_2_H_2_ gases	High quality, large size, monolayer or bilayer graphene	Requires expensive equipment, throughput issue and scaling requires significant effortSome of the gaseous raw materials are hazardous, the use is limited in some applications and a concern for large-scale production	[149,150,151,152,153,154,155,156,157,158,159,160,161,162,163,164,165,166,167,168,169]

**Table 3:** The primary synthesis methods on production of CNTs.

Synthesis Methods	Advantages	Disadvantages	References
Arc discharge	Mass production, SWCNTs and MWCNTs	Multi morphology	[4,11,180,181,182,183]
Laser ablation	SWCNTs yield with a controlling diameter distribution	Not suitable for mass production	[184]
Chemical vapordeposition (CVD)	Mass production	Modification process parameters needed to control SWCNTs diameter distribution and yield	[186,187,188,189,190]
